# Two new species of *Protomicrocotyle* Johnston & Tiegs, 1922 (Monogenea: Protomicrocotylidae) parasites of *Caranx hippos* (Linnaeus, 1776) (Carangiformes: Carangidae), from Tecolutla, Veracruz, Mexico

**DOI:** 10.1590/S1984-29612026035

**Published:** 2026-08-14

**Authors:** Edgar Salvador Ramírez-Cruz, Scott Monks, Norma Leticia Manríquez-Morán, Griselda Pulido-Flores

**Affiliations:** 1 Universidad Autónoma del Estado de Hidalgo UAEH, Instituto de Ciencias Básicas e Ingenierías ICBI, Centro de Investigaciones Biológicas, Laboratorio de Morfología Animal Mineral de la Reforma, Hidalgo, México; 2 Universidad Autónoma del Estado de Hidalgo UAEH, Dirección de Laboratorios, Mineral de la Reforma, Hidalgo, México; 3 University of Nebraska State Museum, Harold W. Manter Laboratory of Parasitology, Lincoln, NE, United States; 4 Universidad Autónoma del Estado de Hidalgo UAEH, Instituto de Ciencias Básicas e Ingenierías ICBI, Centro de Investigaciones Biológicas, Laboratorio de Sistemática Molecular, Mineral de la Reforma, Hidalgo, México

**Keywords:** Monogenea, morphometry, molecular, genetic distance, Neighbor-Joining, Protomicrocotyle, Monogenea, morfometria, molecular, distância genética, Neighbor-Joining, Protomicrocotyle

## Abstract

Two new species of *Protomicrocotyle* are described as parasites of the gill filaments of *Caranx hippos* from the Gulf of Mexico coast, characterized using morphological, morphometric, and molecular methods. Multivariate statistics (principal component and discriminant analyses) were used for the morphometric analyses. Pairwise genetic distances (*p-distances*) and cluster analyses using the Neighbor-joining method were performed using partial sequences of CO1 mtDNA and 28S rDNA of the new species and partial sequences of other species of Protomicrocotylidae from GenBank. *Protomicrocotyle eamsae* sp. nov. differs morphologically from other congeners by the presence of a retractile glandulomuscular organ in the prohaptor, and *Protomicrocotyle pritchardae* sp. nov. differs from other congeners by the body length and by the arrangement, shape, and number of testes. The new species was recovered as a distinct group, separate from other protomicrocotylids, based on CO1 mtDNA and 28S rDNA analyses. The results of the morphometric analysis of 91 morphological variables and the molecular analysis support the assignment of the new species to *Protomicrocotyle* in *Caranx hippos* from Tecolutla, Veracruz, Mexico.

## Resumo

Duas novas espécies de *Protomicrocotyle* são descritas como parasitos dos filamentos branquiais de *Caranx hippos* da costa do Golfo do Mexico, caracterizadas usando métodos morfológicos, morfométricos e moleculares. Estatísticas multivariadas (análises de componentes principais e discriminantes) foram usadas para as análises morfométricas. Distâncias genéticas pareadas (*p-distances*) e análises de agrupamento usando o método Neighbor-joining foram realizadas usando sequências parciais de CO1 mtDNA e 28S rDNA da nova espécie e sequências parciais de outras espécies de Protomicrocotylidae do GenBank. *Protomicrocotyle eamsae* sp. nov. difere morfologicamente de outros congêneres pela presença de um órgão glandulomuscular retrátil no prohaptor, e *Protomicrocotyle pritchardae* sp. nov. difere de outros congêneres pelo comprimento do corpo e pelo arranjo, forma e número de testículos. A nova espécie foi recuperada como um grupo distinto, separado de outros protomicrocotilídeos, com base nas análises de CO1 mtDNA e 28S rDNA. Os resultados da análise morfométrica de 91 variáveis morfológicas e da análise molecular corroboram a atribuição da nova espécie ao *Protomicrocotyle* em *Caranx hippos* de Tecolutla, Veracruz, Mexico.

## Introduction

Monogenea are flatworms that are distinguished by the presence of a specialized attachment structure, the haptor, located at the posterior end of the body. They have a monoxenous life cycle that is limited exclusively to the aquatic environment (Buchmann & Bresciani, 2006; Pulido-Flores, 2024). Typically, they are found on the skin, gills, or fins of fish, both freshwater and marine, although some monogeneans can also infect amphibians and reptiles (Buchmann & Bresciani, 2006; Kearn, 2014; Mendoza-Garfias et al., 2017; Pulido-Flores, 2024). Some hypotheses based on analyses of molecular data suggest that Monogenea is paraphyletic (does not form a monophyletic group) (Brabec et al., 2023; Justine, 1991a, b; Mollaret et al., 1997); however, pending studies that combine morphological and molecular data in a Total Evidence analysis (Kluge, 1998, 2004), we continue to consider Monogenea to be monophyletic (Pulido-Flores, 2024).

Monogeneans encompass an estimated global diversity of 5,000 known species, comprising 716 genera and 63 families (Caira & Littlewood, 2013). In Mexico, this class is represented by the second-highest number of recorded species of helminth, with approximately 313 nominal species and 54 undetermined taxa. Collectively, these records correspond to 162 genera included in 29 families (García-Prieto et al., 2014; Mendoza-Garfias et al., 2017).

Monogeneans assigned to Protomicrocotylidae are parasites on the gill arches of marine fish, particularly carangid fish (Caballero y Caballero & Bravo-Hollis, 1965, 1967; Kritsky et al., 2011; Ramalingam, 1960; Yamaguti, 1963). Currently, the family is represented by nine genera: *Abortipedia* Unnithan, 1962, *Bilaterocotyle* Chauhan, 1945, *Bilaterocotyloides* Ramalingam, 1961, *Chauhanocotyle* Khoche & Dad, 1975, *Lethacotyle* Manter & Price, 1953, *Neomicrocotyle* Ramalingam, 1960, *Protomicrocotyle* Johnston & Tiegs, 1922, *Vallisiopsis* Subhapradha, 1951, and *Youngiopsis* Lebedev, 1972, with approximately 43 species assigned to the family (Lebedev, 1986; Ramírez-Cruz et al., 2023; WoRMS, 2026).

All known species of *Protomicrocotyle* are parasites of fish belonging to the family Carangidae Nelson, 1984, with the majority having been reported as parasites of the genera *Caranx* Lacepède, 1801 (Boada et al., 2012; Bravo-Hollis, 1966, 1979; Caballero y Caballero & Bravo-Hollis, 1965; Hargis, 1957; Iruegas-Buentello, 1999; Kritsky et al., 2011; Luque & Alves, 2001; Mendoza-Garfias et al., 2017; Montoya-Mendoza et al., 2017; Ramalingam, 1960; Ramírez-Cruz et al., 2023; Vianna et al., 2020; Violante-González et al., 2019; Wahl, 1972; Yamaguti, 1953); a single species was described from *Trachinotus* Lacepède. To date, 10 species have been described: *P. mirabilis* (MacCallum, 1918) Johnston & Tiegs, 1922 (type species) from the Atlantic Ocean in *C. hippos* (Linnaeus) from the New York Aquarium (MacCallum, 1918); *P*.*manteri* Bravo-Hollis, 1966 from La Paz, Baja California Sur, Mexico, in *Trachinotus paitensis* Cuvier (= *T. paloma* Jordan & Starks) (Bravo-Hollis, 1966); *P*.*nayaritensis* Bravo-Hollis, 1979 from Isla Isabel, Nayarit, Mexico, in *C.* *caninus* Günther, (originally identified as *C*.*hippos* *caninus*) (Bravo-Hollis, 1979); *P*.*ivoriensis* Wahl, 1972 from Ebrié Lagoon, Ivory Coast, Western Africa, in *C. hippos* (Wahl, 1972); *P*.*celebesensis* Yamaguti, 1953 from Celebes Island, Indonesia, from *Caranx* sp. (Yamaguti, 1953); *P*.*madrasensis* Ramalingam, 1960 in *C*. *affinis* Rüppell; *P*.*mannarensis* Ramalingam, 1960; *P*.*minutum* Ramalingam, 1960 in *C*.*sexfasciatus* Quoy & Gaimard from India (Ramalingam, 1960); *P*.*carangis* Pillai & Pillai, 1978 from Kerala coast, India, in *C. ignobilis* (Forsskål) [= *C*.*sansun* (Fabricius)] (Pillai & Pillai, 1978); and the recently described species, *P. veracruzensis* Ramírez-Cruz, Monks, Manríquez-Morán, Violante-González & Pulido-Flores, 2023 from Casitas and Puerto de Veracruz, Veracruz, Mexico, in *C*.*latus* Agassiz (Ramírez-Cruz et al., 2023).

As part of an ongoing studies of the biodiversity of monogenean parasites of marine fish from the Gulf of Mexico, two new species of *Protomicrocotyle* were found on the gill filaments of *C. hippos* in the locality of Tecolutla, Veracruz. The new species are illustrated and described herein using morphological, morphometrics and molecular data.

## Material and Methods

### Specimen collection

**Figure 1:**
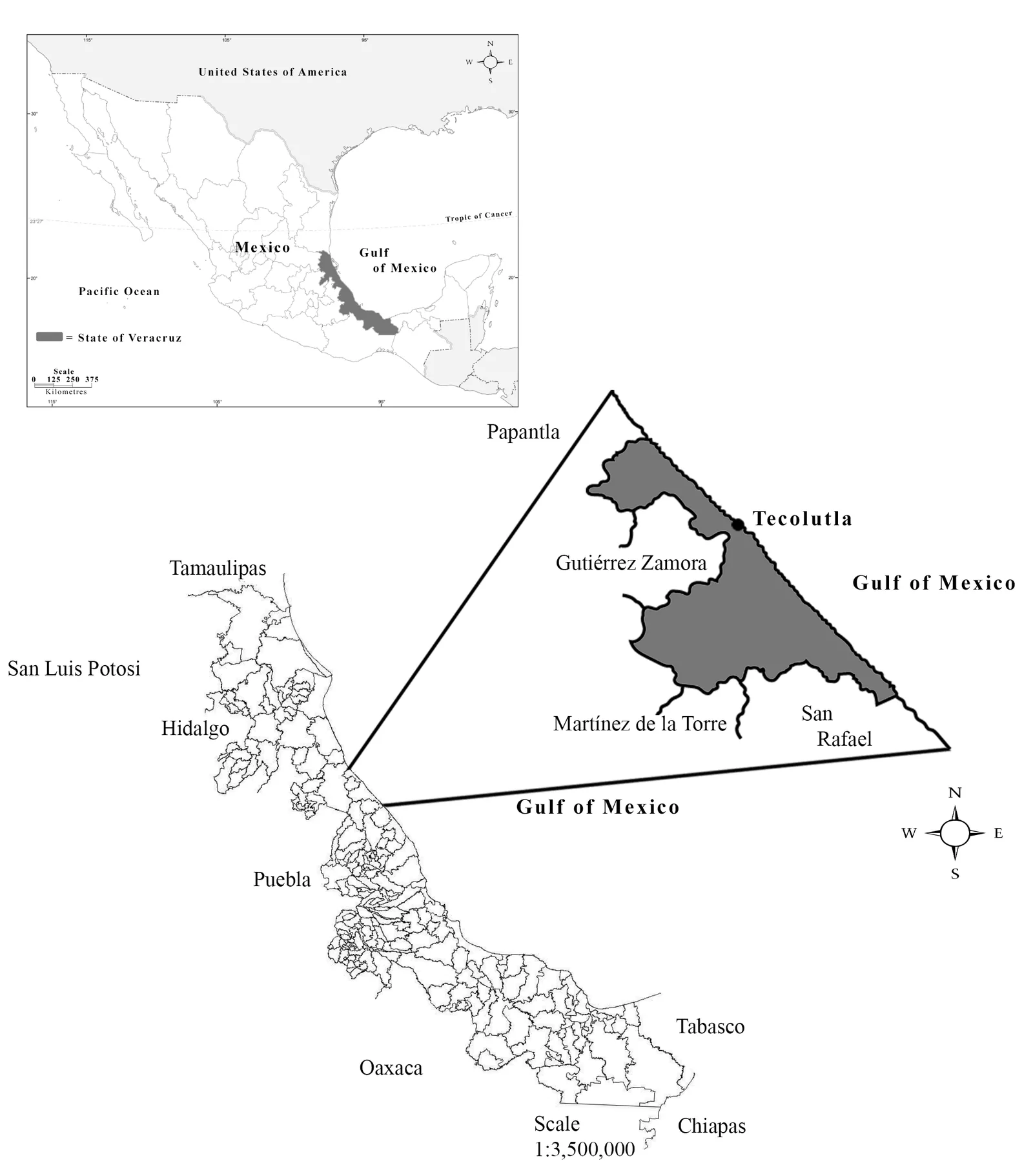
Geographic location of the sampled locality (bold point) in the state of Veracruz, Mexico.

Fourteen specimens of *Caranx hippos* were collected (November 2021) from the coastal waters off Tecolutla, Veracruz (20° 28′ 39″ N; 97° 00′ 30″ W) (Figure 1). Fish were obtained through commercial purchases from local fisherman. Each fish was sexed, measured, and photographed in order to facilitate taxonomic identification, in accordance with the recommendations set forth in the specialized literature (Nelson, 2006). Fish were maintained on ice until necropsied.

Necropsies were carried out in the field. Gill arches were extracted from the gill cavity of each fish, placed in a labeled bag with sea water, and kept on ice pending examination. Each branchial arch was examined using a Leica Zoom 2000 stereoscopic microscope. Monogeneans were collected from the gill filaments and water in each bag was examined for detached worms. Specimens for morphological study were relaxed with hot water and stored in A.F.A. (Lamothe-Argumedo, 1997; Pritchard & Kruse, 1982), and specimens for molecular analyses were stored at 100% ethanol.

Type material and vouchers of the new species were deposited in the Colección Nacional de Helmintos (CNHE), the Harold W. Manter Laboratory of Parasitology Collection (HWML), University of Nebraska-Lincoln, and the Colección de Helmintos (CHE) of the Centro de Investigaciones Biológicas of the Universidad Autónoma del Estado de Hidalgo (UAEH).

### Morphological study

Specimens of *Protomicrocotyle* were stained using Gomori’s trichrome, Mayer’s carmalum, or Delafield’s hematoxylin, dehydrated in an ethanol series, cleared in methyl salicylate, and mounted individually as whole mounts on slides in Canada balsam. Specimens were identified based on morphological keys and specialized literature (Bravo-Hollis, 1979; Kritsky et al., 2011; Ramalingam, 1960; Ramírez-Cruz et al., 2023; Yamaguti, 1963). The protocol for morphological measurements followed Ramírez-Cruz et al. (2023).

Specimens were examined using a Leica DM LB2 compound optical binocular microscope equipped with differential interference contrast optics; measurements were taken using a calibrated ocular micrometer and presented in micrometers (*µm*) in the following format: minimum, maximum, and in parentheses, mean, standard deviation, and the number of individuals or anatomical structures that were measured (n). Description of the shape of anatomical structures was based on Clopton (2004). Line drawings were made using a drawing tube and processed in Adobe® Illustrator® CS6 2012 and Adobe Photoshop® CS6 2012 software (Adobe® Systems Inc., San Jose, California). Photographs were taken using a Digital Microscope VHX-7000 series (Unidad Central de Laboratorios, UAEH).

### Morphometric analyses

For comparative purposes, some type, paratypes and vouchers specimens from the CNHE, HWML and CHE were examined: *Protomicrocotyle mirabilis* (CNHE-12822–12825, HWML-216985–216994, CHE-P00147); *P. manteri* (CNHE-21; 134; 344; 345; 346; 347; 348; 3114; 3115), *P. nayaritensis* (CNHE-158; 159), *P. veracruzensis* (holotype CNHE-12819; paratypes CNHE-12820–12821, HWML-216978–216984 and 2166998, CHE-P00148), *Neomicrocotyle pacifica* (Meserve, 1938) Yamaguti, 1968 (CNHE: 371; 372; 3116).

A total of 91 morphological variables from 117 specimens of *Protomicrocotyle* were analyzed (88 continuous and three meristic). The data matrix was integrated with the data of the aforementioned species and that of the newly identified species. For analysis, the quantitative variables (continuous and meristic) were transformed into the Natural logarithm (*Ln*). Multivariate analyses included Principal Component Analyses (PCA) and Linear Discriminant Analyses (LDA). The PCA is a standard method for reducing the dimensionality of morphometric and ecological data (Hammer et al., 2001). In this study, the eigenvalues and eigenvectors of the variance-covariance matrix were used for reduction of dimensionality. The variance-covariance matrix was used because all variables were measured with the same metric unit (*µm* transformed to *Ln*). The weighting coefficients (loadings table = component loadings) were used to determine the relationship and influence that each original variable had on the principal components. The scree plot was used to visualize the "breaking point" where the components stop providing meaningful information and the convex hulls tool is activated in the scatter plot to automatically enclose points from the same group within a polygon, which facilitates the observation of the groups.

The new variables are linear combinations of the original variables and were used for the discriminant analyses. The variables that did not contribute information were eliminated, and the Linear Discriminant Analysis (LDA) was performed with the new matrix. A total of 55 morphological variables, identified from 117 specimens of *Protomicrocotyle*, were analyzed. The matrix with the transformed data was used. The variance-covariance matrix was used for standard procedure of the LDA (Hammer et al., 2001). The distribution of groups was visualize using the scatter plot of the convex hulls. The original resubstituting matrix and the Jackknife Matrix (*Cross-Validation*) were used to determine the original accuracy percentage (% Correctly Classified [% CC]) and to verify the robustness of the statistical model (Hammer et al., 2001). The PCA and LDA analyzes were performed in PAST v.4.17 (Hammer et al., 2001). Plots were edited for publication in Adobe® Photoshop®.

### DNA extraction, amplification and sequencing

DNA extraction and amplification were carried out on specimens collected in the field during 2021 and identified as species of *Protomicrocotyle*. Whole genomic DNA was extracted using a DNeasy Blood and Tissue Kit (Qiagen, Hilden, Germany) following the manufacturer’s protocol. Fragments of CO1 mtDNA were amplified using the primers JB3-F (ASmit1) (5’-TTTTTTGGGCATCCTGAGGTTTAT-3’) and JB4-R (ASmit2) (5’-TAAAGAAAGAACATAATGAAAATG-3’) (Bowles et al., 1993; Ramírez-Cruz et al., 2023; Tambireddy et al., 2016). The primers LSU5-F (5’-TAGGTCGACCCGCTGAAYTTAAGC-3’) and EC-D2-R (5’-CCTTGGTCCGTGTTTCAAGACGGG-3’) were used for 28S rDNA amplification (Littlewood et al., 1997; Ramírez-Cruz et al., 2023; Tkach et al., 2003).

Polymerase Chain Reactions (PCR) were performed in a total of 25 µL solution consisting of 4 µL of template DNA, 4.85 µL of master solution [0.15 µL of Taq DNA polymerase (5 µ/mL; BioTecMol), 1 µL dNTPs (2.5 mM; Promega), 0.2 µL of each primer (10 nM), 1.8 µL of 5x PCR buffer (BioTecMol), 1.5 µL of MgCl_2_ (25 mM; BioTecMol)], and 16.15 µL of distilled water. The cycling conditions for CO1 mtDNA and 28S rDNA included initial denaturation at 94°C for 5 min, followed by 38 cycles of 94°C for 30 s, 45°C for 30 s, and 72°C for 1:10 min, and a final extension of 7:00 min at 72°C.

The PCR products were visualized using an electrophoresis agarose gel and were purified using a polyethylene glycol (PEG) protocol for purification of the DNA and removal of residual primers, dNTPs, and other superfluous products (Monzalvo et al., 2024; Ramírez-Cruz et al., 2023; Schmitz & Riesner, 2006). The PCR products were transferred to a 1.5 mL microtube containing 20 µl of 20% PEG solution (PEG 2.5 M NaCl) and incubate at 37°C for 10 minutes. The PCR products were then centrifuged at 14,000 revolutions per minute (r.p.m.) for 10 minutes, the supernatant solution was removed with a micropipette (avoid contact with the DNA button), 150 µl of cold (-20°C) 95% ethanol was added, and the tube was centrifuged again at 14,000 r.p.m. for 10 minutes. The supernatant solution was removed with a micropipette, avoiding contact with the DNA button. The microtubes was placed in a DNA concentrator (Savant SC110A Speedvac) at 50–60 °C for approximately 10 minutes, until all the ethanol had evaporated. The DNA button was resuspended in 100 µl of distilled water. Finally, the quality of the sample was evaluated using electrophoresis on an agarose gel (Alejos-Velázquez et al., 2014; Lis, 1980).

Samples were sequenced using a BigDye Terminator v3.1 Cycle Sequencing Kit in 10 µL reactions, and a 3730xl DNA Analyzer (Thermo Fisher Scientific), using the JB3-F, and LSU5-F primers for CO1 mtDNA and 28S rDNA, respectively. Sequencing was performed at the Instituto de Biología, UNAM, Mexico. Sequence data and electropherograms were inspected and edited using Pregap4 and Gap4 modules by Staden software V.1.6 (Staden, 1996).

### Alignment, genetic distances and the Neighbor-Joining tree

Partial sequences obtained for the CO1 mtDNA and the 28S rDNA regions were aligned with sequences from other species of *Protomicrocotyle* and of *Allodiscocotyla diacanthi* Unnithan, 1962 (Allodiscocotylidae; outgroup) retrieved from GenBank (Table 1). Pairwise genetic distances (*p-distance*) between all sequences were calculated in MEGA 11 (Tamura et al., 2021) (Table 1). A distance matrix was used for clustering analysis and the presentation of tree topology. The Neighbor-Joining (NJ) method was used to builds a tree from a matrix of pairwise evolutionary distances relating to the set of taxa being studied, therefore, the algorithm of the method finds the pairs of sequences that minimize the total length of the topology of the tree in each iteration (Gascuel & Steel, 2006; Saitou & Nei, 1987). The NJ analyses from CO1 mtDNA and 28S rDNA was performed in MEGA 11 with bootstrap analysis based on 1,000 resampling of each data set (Tamura et al., 2021). Trees were edited in Adobe® Photoshop®.

**Table 1:** List of monogeneans included in the genetic distances analyses and Neighbor Joining analysis. For each sequence, the GenBank accession number of partial sequences of cytochrome *b* and the conserved ribosomal regions of the *28S rDNA* is given. New sequences obtained in the present study are in bold.

**Species of monogeneans**	**Host**	**Genbank ID**		**Reference**
		**CO1 mtDNA**	**28S rDNA**	
**Protomicrocotylidae**				
*Protomicrocotyle eamsae* sp. nov.	*Caranx hippos*	**PZ380651**	**PZ377455**	This study
		**PZ380652**	**PZ377456**	
		**PZ380653**	**PZ377457**	
		**PZ380654**	**PZ377458**	
*Protomicrocotyle pritchardae* sp. nov.		**PZ380648**	**PZ377459**	This study
		**PZ380649**	**PZ377460**	
		**PZ380650**	**PZ377461**	
		**-**	**PZ377462**	
*Protomicrocotyle mirabilis* (MacCallum, 1918) Johnston & Tiegs, 1922		OR282823	OR282885	Ramírez-Cruz et al. (2023)
		OR282824	OR282887	
		OR282831	OR282888	
		OR282832	OR282892	
*Protomicrocotyle veracruzensis* Ramírez-Cruz, Monks, Manríquez-Morán, Violante-González & Pulido-Flores, 2023	*Caranx latus*	OR282833	OR282895	Ramírez-Cruz et al. (2023)
		OR282834	OR282896	
		OR282835	OR282897	
		OR282836	OR282898	
*Neomicrocotyle pacifica* (Meserve, 1938) Yamaguti, 1968	*Caranx caballus*	–	AF382043	Olson & Littlewood (2002)
*Neomicrocotyle* sp.	*Caranx sexfasciatus*	–	KF378589	Justine et al. (2013)
*Lethacotyle vera* Justine, Rahmouni, Gey, Schoelinck & Hoberg, 2013*	*Caranx papuensis*	–	KF378588	
*Bilaterocotyloides carangis* Ramalingam, 1961	*Megalaspis cordyla*	KF804043	–	Tambireddy et al. (2016)
		–	KJ201022	
*Bilaterocotyloides madrasensis* Radha, 1966	*M*. *cordyla*	KF804041	KF804029	
		–	KF804037	
**Outgroup**				
**Allodiscocotylidae**				
*Allodiscocotyla diacanthi* Unnithan, 1962	*Scomberoides commersonnianus*	KF804045	KF804033	
		KF804046	KF804038	

* Reported as *Lethacotyle* sp. in GenBank.

GenBank ID numbers in **bold** corresponds to the sequences generated in the present study.

## Results

### Morphology study

Class Monogenea van Beneden, 1858

Subclass Polyopisthocotylea Odhner, 1912

Order Mazocraeidea Bychowsky, 1937

Family Protomicrocotylidae Johnston & Tiegs, 1922

Genus *Protomicrocotyle* Johnston & Tiegs, 1922

*Protomicrocotyle eamsae* sp. nov.

**Figure 2:**
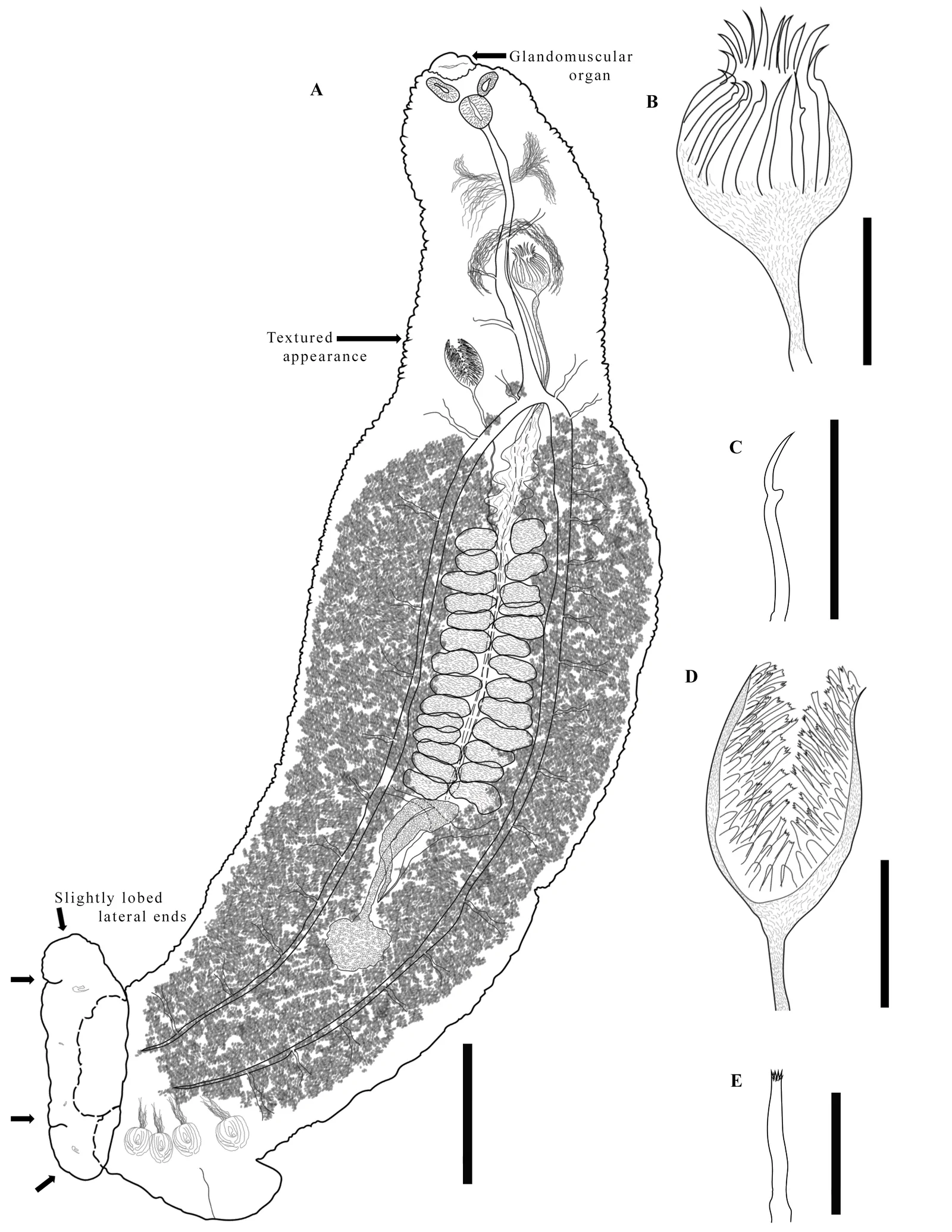
Line drawings of the holotype (CNHE 12575) of *Protomicrocotyle eamsae* sp. nov. from the gills of *Caranx hippos*. (A) Complete body, ventral view. The arrows indicate the slightly lobed lateral ends of the haptoral lappet. Bar 200 μm; (B) Male copulatory organ. Bar 50 μm; (C) Spine of MCO. Bar 50 μm; (D) Vaginal vestibule. Bar 50 μm; (E) Spine of vaginal vestibule. Bar 25 μm.

**Figure 3:**
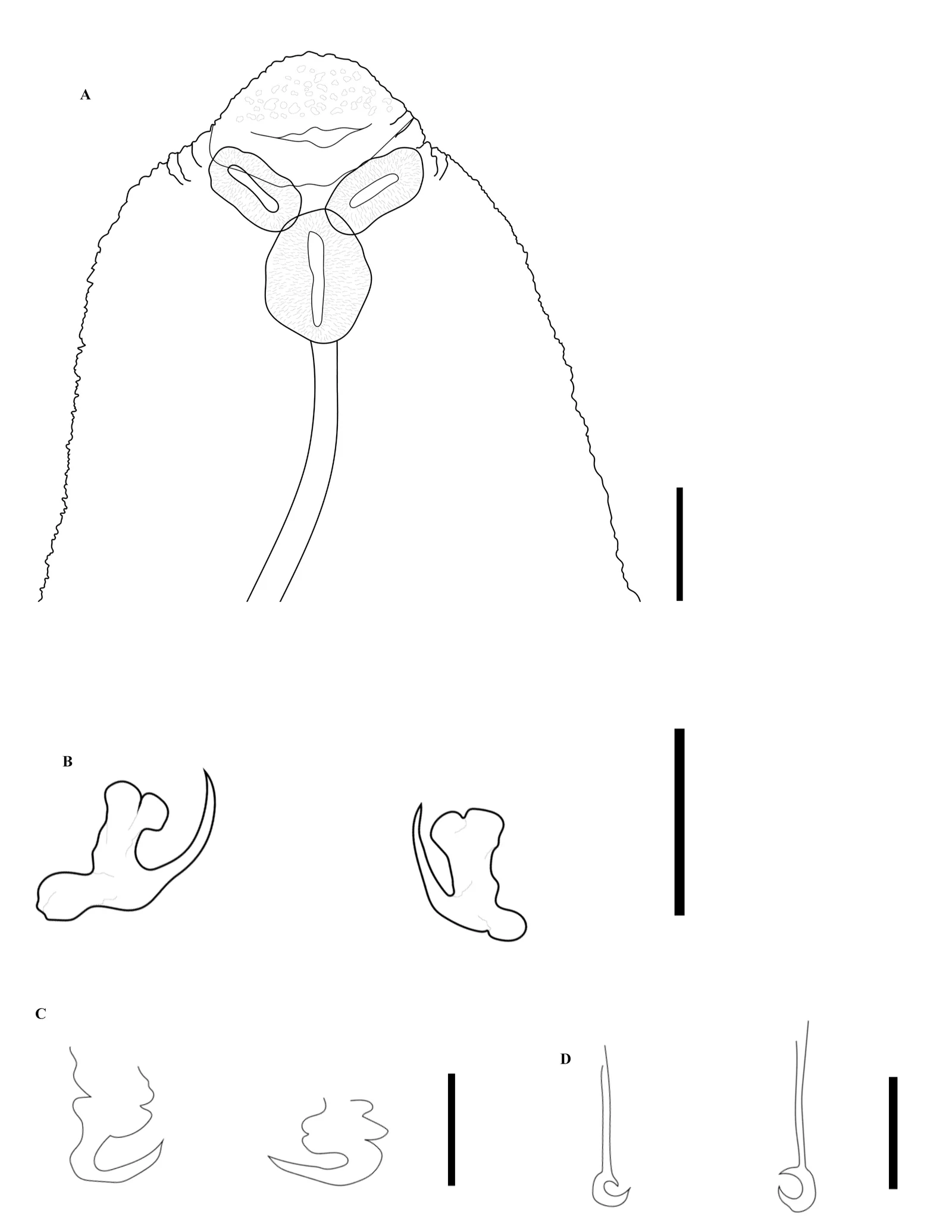
Line drawings of the paratype (CNHE 12576) of *Protomicrocotyle eamsae* sp. nov. (A) Details of the anterior region, ventral view. Bar 50 μm; (B) Lateral anchors. Bar 16 μm; (C) Median anchors. Bar 16 μm; (D) Hooks. Bar 16 μm.

**Figure 4:**
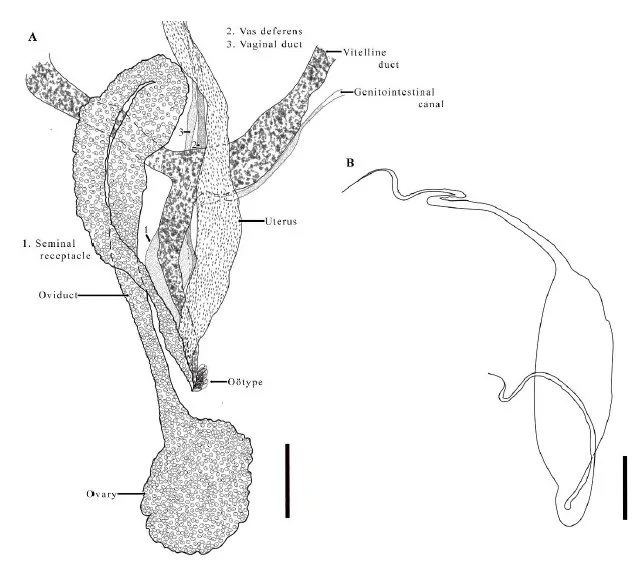
(A) Reproductive organs of *Protomicrocotyle eamsae* sp. nov. (CNHE 12575) Bar 50 μm; (B) Egg with two long polar filaments (Paratype CNHE 12576). Bar 50 μm.

**Figure 5:**
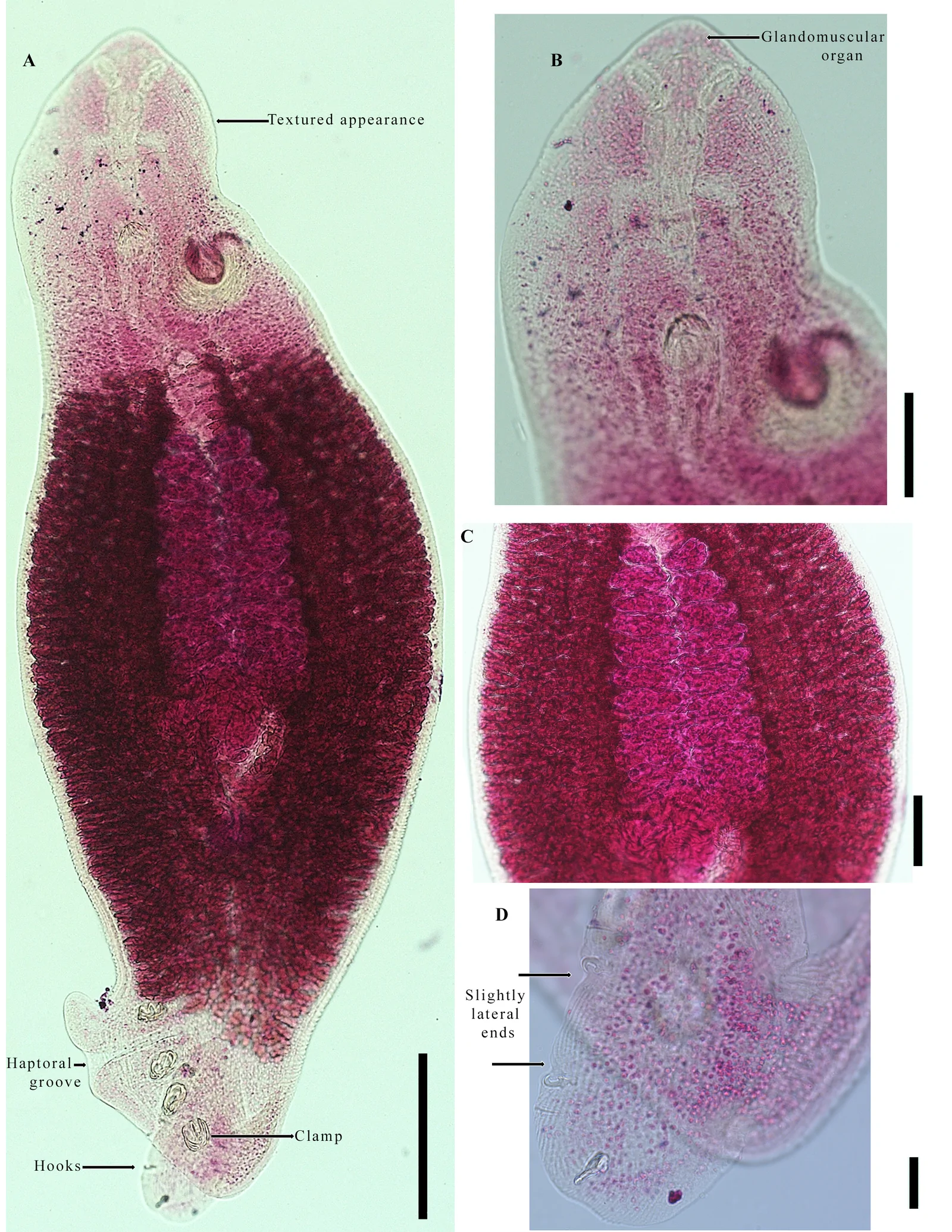
Light micrographs of the paratype (HWML 218464) of *Protomicrocotyle eamsae* sp. nov. (A) Complete body, ventral view. Bar 250 μm; (B) Details of the prohaptor, ventral view. Bar 100 μm; (C) Body trunk, ventral view. Bar 100 μm; (D) Details of the haptoral lappet. Bar 25 μm. The arrows indicate the slightly lobed lateral ends of the haptoral lappet. Mayer’s Carmalum stain.

**Figure 6:**
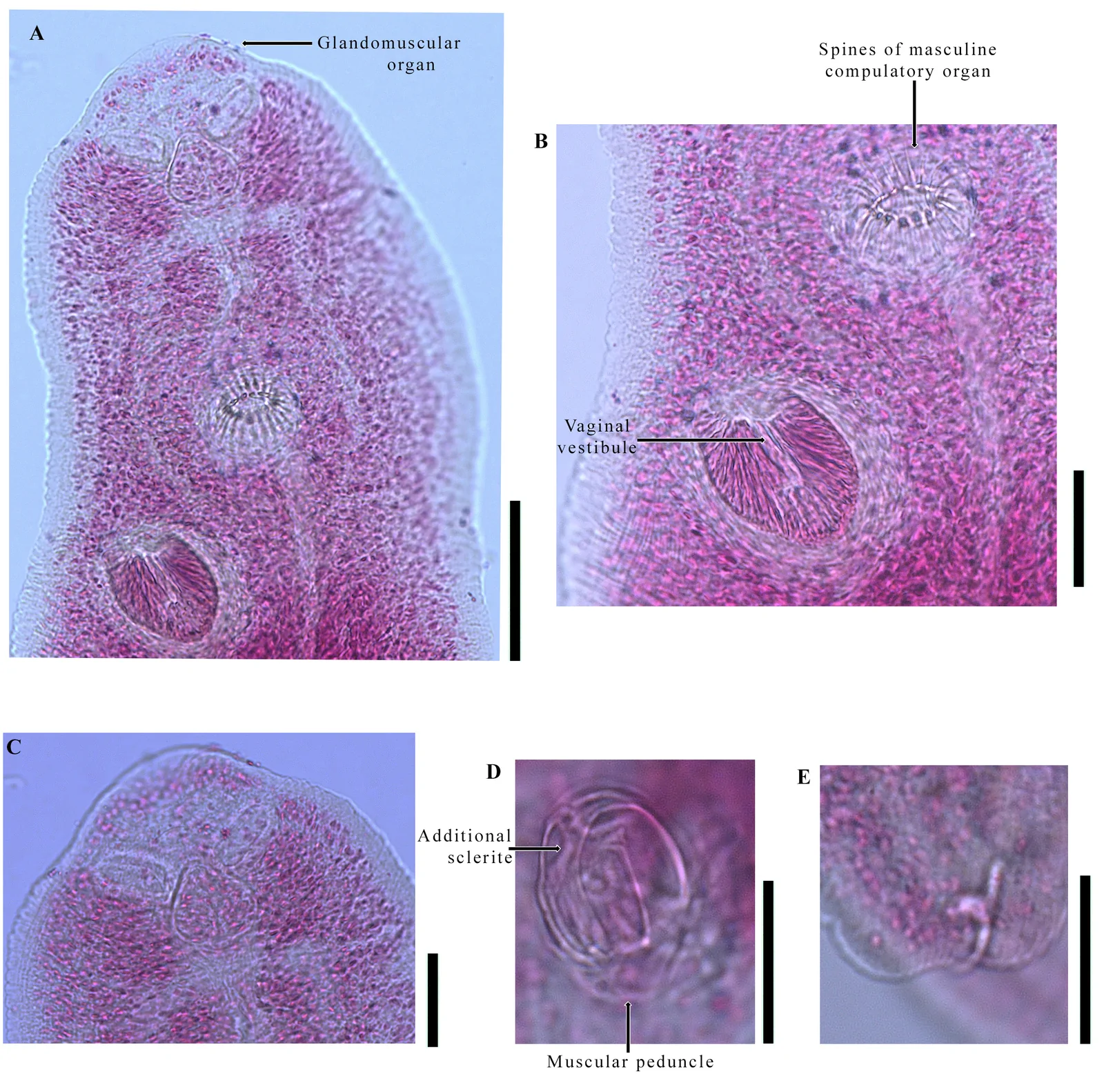
Light micrographs of the paratype (HWML 218465) of *Protomicrocotyle eamsae* sp. nov. (A) Anterior region, ventral view. Bar 100 μm; (B) Details of the masculine copulatory organ and vaginal vestibule. Bar 50 μm; (C) Aperture oral and pseudo-suckers. Bar 50 μm; (D) Clamp. Bar 50 μm; (E) Lateral anchor. Bar 50 μm. Mayer's Carmalum stain.

*Description* (Figures 2, 3, 4, 5, 6; Tables 2, 3)*.* Based on eight specimens, stained and mounted on slides to be viewed from ventral side of the body. Measurements and data of other species are presented in Tables 2 and 3.

**Table 2:** Measurements (in µm) of haptoral armature (anchors, and hooks) of species of *Protomicrocotyle* from the Gulf of Mexico.

**Species**	***Protomicrocotyle eamsae* sp. nov.**	***Protomicrocotyle pritchardae* sp. nov.**	***Protomicrocotyle mirabilis***	***Protomicrocotyle veracruzensis***
Locality	Tecolutla, Veracruz	Tecolutla, Veracruz	Veracruz, Mexico	Casitas and Puerto de Veracruz, Veracruz, Mexico
Host	*Caranx hippos*	*C. hippos*	*C. hippos*	*Caranx latus*
Site	Gills filaments	Gills filaments	Gills filaments	Gills filaments
Anchor/Hook	μm	μm	μm	μm
Right lateral anchor (Ac1)				
Total length	46 (43–50, n = 7)	54 (50–60, n = 7)	49 (38–70 ± 8, n= 26)	43 (38‒50, n = 24)
Total width	5 (4–5, n = 7)	5 (5–6, n = 6)	5 (5–7, n = 26)	5 (5‒7, n = 24)
Opening length	9 (7–10, n = 7)	7 (n = 4)	10 (7–13, n = 24)	10 (7‒13, n = 24)
Internal root length	13 (11–14, n = 7)	14 (13–18, n = 7)	16 (11–20, n = 26)	16 (11‒19, n = 24)
Internal root width	6 (5–6, n = 7)	7 (6–8, n = 5)	7 (5–10, n = 27)	7 (5‒10, n = 24)
External root length	21 (11–24, n = 7)	26 (23–28, n = 7)	23 (18–29, n = 26)	16 (13‒20, n = 24)
External blade length	11 (8–12, n = 7)	12 (8–13, n = 5)	15 (12–20, n = 27)	23 (18‒29, n = 24)
Internal blade length	20 (17–22, n = 7)	21 (17–23, n = 7)	19 (10–25, n = 26)	20 (16‒25, n = 24)
Point length	12 (10–14, n = 7)	16 (11–20, n = 7)	14 (15–19, n = 26)	13 (5‒19, n = 24)
Left lateral anchor (Ac1’)				
Total length	51 (46–56, n = 7)	54 (52–58, n = 7)	50 (40–64, n = 29)	43 (38‒50, n = 24)
Total width	5 (4–6, n = 7)	5 (5–6, n = 7)	6 (5–7, n = 28)	5 (5‒7, n = 24)
Opening length	10 (8–12, n = 7)	7 (4–10, n = 8)	10 (7–16, n = 25)	10 (7‒13, n = 23)
Internal root length	13 (12–14, n = 7)	15 (13–18, n = 8)	16 (11–20, n = 29)	15 (11‒17, n = 24)
Internal root width	5–7 (6 ± 1, n= 7)	7 (6–8, n = 8)	6 (5–8, n = 29)	7 (5‒8, n = 24)
External root length	10–13 (12 ± 1, n= 7)	11 (8–14, n = 8)	14 (10–19, n = 28)	15 (12‒19, n = 24)
External blade length	22–25 (23 ± 1, n= 7)	25 (23–28, n = 8)	24 (17–34, n = 29)	24 (17‒34, n = 24)
Internal blade length	17–20 (19 ± 1, n= 7)	20 (19–22, n = 8)	20 (13–31, n = 29)	20 (13‒31, n = 24)
Point length	12–14 (13 ± 1, n= 7)	14 (10–17, n = 8)	15 (8–24, n = 29)	15 (8‒24, n = 24)
Right medial anchor (Ac2)				
Total length	26 (24–28, n = 6)	28 (25–31, n = 8)	26 (24–31, n = 26)	27 (25‒29, n = 20)
Total width	2 (2–4, n = 6)	3 (2–4, n = 8)	4 (2–5, n = 26)	4 (2‒5, n = 22)
Opening length	4 (4–5, n = 6)	4 (2–6, n = 6)	5 (4–8, n = 24)	5 (4‒8, n = 22)
Internal root length	5 (4–6, n = 6)	5 (4–7, n = 6)	6 (4–11, n = 23)	7 (5‒11, n = 22)
Internal root width	2 (n = 6)	3 (2–4, n = 5)	3 (2–4, n = 23)	3 (2‒4, n = 22)
External root length	5 (4–7, n = 6)	6 (4–8, n = 6)	7 (4–13, n = 23)	9 (7‒13, n = 22)
External blade length	16 (14–17, n = 6)	18 (16–20, n = 6)	16 (11–19, n = 24)	16 (13‒19, n = 22)
Internal blade length	12 (11–13, n = 6)	16 (12–18, n = 6)	13 (8–17, n = 23)	13 (11‒17, n = 22)
Point length	9 (7–12, n = 6)	12 (10–13, n = 6)	10 (5–14, n = 26)	9 (5‒12, n = 22)
Left medial anchor (Ac2’)				
Total length	26 (25–29, n = 6)	27 (25–29, n = 6)	28 (23–32, n = 26)	27 (24‒29, n = 20)
Total width	3 (2–4, n = 6)	4 (2–4, n = 5)	4 (2–4, n = 26)	3 (2‒4, n = 20)
Opening length	3 (1–4, n = 6)	4 (2–5, n = 4)	5 (4–11, n = 24)	6 (4‒11, n = 20)
Internal root length	3 (2–4, n = 6)	5 (2–6, n = 4)	7 (2–12, n = 24)	9 (7‒12, n = 20)
Internal root width	2 (n = 6)	3 (2–4, n = 4)	3 (2–5, n = 23)	3 (2‒5, n = 20)
External root length	5 (5–6, n = 6)	5 (4–5, n = 4)	7 (4–11, n = 24)	9 (7‒11, n = 20)
External blade length	16 (14–17, n = 6)	19 (17–19, n = 4)	17 (12–20, n = 25)	18 (14‒20, n = 20)
Internal blade length	13 (12–14, n = 6)	16 (14–17, n = 4)	14 (8–20, n = 25)	15 (12‒20, n = 20)
Point length	9 (6–11, n = 6)	13 (12–13, n = 4)	11 (8–14, n = 26)	11 (8‒13, n = 20)
Right central hook (Ho)				
Total length	14 (14–16, n = 8)	16 (13–18, n = 11)	17 (12–19, n = 30)	18 (17‒19, n = 22)
Total width	-	2 (n = 3)	1–2 (2, n = 30)	2 (2‒2, n = 22)
Left central hook (Ho’)				
Total length	15 (13–17, n = 8)	17 (16–19, n = 9)	17 (13–19, n = 26)	18 (17‒19, n = 22)
Total width	-	2 (n = 3)	1–2 (2, n = 26)	2 (2‒2, n = 22)
References	This study	This study	This study	Ramírez-Cruz et al. (2023)

**Table 3:** Comparative measurements of species of *Protomicrocotyle* from Mexico and USA.

**Species**	***Protomicrocotyle eamsae* sp. nov.**	***Protomicrocotyle pritchardae* sp. nov.**	***P. veracruzensis***	***P. mirabilis***	***P. mirabilis***	***P. manteri***	***P. nayaritensis***
Locality	Tecolutla, Veracruz, Mexico	Tecolutla, Veracruz, Mexico	Casitas and Puerto de Veracruz, Veracruz, Mexico	Veracruz, Mexico	Florida Bay, USA	La Paz, Baja California Sur, Mexico	Isla Isabel, Nayarit, Mexico
Host	*Caranx hippos*	*C. hippos*	*C. latus*	*C. hippos*	*C. hippos*	*Trachinotus paitensis*	*C. caninus*
Site	Gill filaments	Gill filaments	Gill filaments	Gill filaments	Gill filaments	Gill filaments	Gill filaments
	µm	µm	µm	µm	µm	µm	µm
Total length	1682 (1135‒1879)	4117 (3721–4380)	3858 (3209‒4709)	2861 (805–4673)	2240 (1470‒3080)	1360‒3680	6820‒8580
Total width	474 (390–622)	537 (488–573)	804 (586‒1098)	295 (134–537)	322 (214‒449)	400‒800	770‒1990
Haptor width	307 (275–350)	438 (410–470)	575 (415‒980)	255 (70–425)	161	590**	1050
Right side of haptoral lappet length	100 (72–113)	174 (145–187)	173 (136‒240)	117 (60–217)	64**	136**	250**
Left side of haptoral lappet length	105 (90–126)	169 (124–198)	194 (124‒259)	120 (40–202)	72**	121**	150**
Central haptoral lappet length	114 (82–131)	196 (170–220)	206 (164‒259)	125 (40–214)	88**	181**	300**
Haptoral lappet width	337 (295–375)	726 (645–820)	769 (580‒920)	437 (105–785)	241	348**	643‒801
Clamps length	37 (33–40)*	45 (41–48)*	64 (47‒74)*	45 (14‒65)*	57 (49‒67)	50‒55	45‒48
Number of spines in MCO	24 (18–26)	21 (13–35)	23 (16‒28)	22 (16–29)	19 (16‒21)	33‒38	48‒54
Number of spines in vaginal vestibule	52 (43–58)	45 (35–61)	49 (33‒59)	45 (31–65)	43	15	22‒46
Number of testes	31 (27–34)	60 (53–67)	47 (36‒69)	31 (21–38)	27 (23‒33)	22‒33	46‒48
Testes length	35 (23–43)	53 (43–66)	47 (31‒59)	45 (17–79)	43 (28‒54)	60.2	88
Testes width	89 (66–109)	82 (65–102)	142 (96‒176)	50 (14–86)	43 (28‒54)	116.5	129
Genital pore to anterior end distance	257 (190–350)	552 (500–625)	506 (340‒615)	370 (140–660)	250	289‒471	858‒1001
Vaginal opening to anterior end distance	346 (270–445)	690 (635–755)	610 (440‒730)	464 (175–740)	330	84‒155	1072‒1258
Reference	This study	This study	Ramírez-Cruz et al. (2023)	Ramírez-Cruz et al. (2023)	Kritsky et al. (2011)	Bravo-Hollis (1966)	Bravo-Hollis (1979)

* Data obtained from the average length of the four clamps;

** Data obtained from the drawings of the article of each species.

*Body* (Figure 2A, 5A). Body fusiform; anterior end conical. Body length (including the haptor) 1135–1879 (1682 ± 213, n = 8); width 390–622 (474 ± 72, n = 8) (Figure 2A, 5A, 5C). Tegument with striations along body surface, imparting a textured appearance, more obvious in the anterior region (Figures 2A, 5A, 6A). Paired prohaptoral suckers anterolateral, septate, muscular, oblique, and very shallowly dolliform; right sucker 36–50 (42 ± 6, n = 8) long, 23–30 (27 ± 2, n = 8) wide; left sucker 37–53 (43 ± 5, n = 8) long, 24–31 (28 ± 3, n = 8) wide (Figures 2A, 3A, 5A, 5B, 6A, 6C). Aperture oral ventral, subterminal, finely ovoid, 25–29 (26 ± 2, n = 6) long, 20–28 (24 ± 3, n = 6) wide. Glandomuscular organ (*sensu*Bravo-Hollis, 1966) present (Figures 3B, 5A, 5B, 6A, 6C). Pre-pharynx absent; pharynx broadly elliptoid, muscular, 30–51 (41 ± 5, n = 8) long, 38–48 (41 ± 3, n = 8) wide (Figures 2A, 3A, 6A, 6C). Esophagus 320 (n = 1) long and 20 (n = 1) wide, with lateral diverticula. Cecal bifurcation posterior to male copulatory organ (MCO), 470 (n = 1) from anterior end of worm. Ceca lateral to midline, extend posteriorly to haptoral region but failing to reach haptoral lappet; cecal diverticula present that extend to margins of body (Figures 2A, 5A).

*Haptor* (Figures 2A, 5A, 5D). Haptor asymmetrical, armed with a row of four gastrocotylid-type clamps, each having a short muscular peduncle; haptoral groove between first and second clamps (Figures 5A, 2A, 6D). Haptor 245–430 (334 ± 55, n = 8) long, 275–350 (307 ± 30, n = 8) wide at the level of second clamp (Figure 5A). Clamps similar in size: first clamp 29–50 (39 ± 9, n = 6) long by 36–48 (42 ± 5, n = 6) wide; second clamp 28–44 (33 ± 4, n = 5) long by 41–48 (45 ± 3, n = 5) wide; third clamp 28–49 (40 ± 9, n = 6) long by 37–53 (46 ± 6, n = 6) wide; and fourth clamp 28–47 (39 ± 7, n = 7) long by 37–55 (45 ± 8, n = 7) wide (Figure 2A, 6D). Haptoral lappet transversely elongate with slightly lobed lateral ends (Figures 2A, 5D); right lateral length 72–113 (100 ± 6, n = 7), left lateral length 90–126 (105 ± 12, n = 7), and central length 82–131 (114 ± 16, n = 7). Haptoral lappet 295–375 (337 ± 24, n = 8) wide, armed with two pairs of anchors and one pair of hooks (Figures 3B, 3C, 3D, 5D, 6E; Table 2).

*Male reproductive structures* (Figures 2B, 5B, 6A, 6B). Testes intercecal, 27–34 (31± 3, n = 6) in number, very shallowly doliform in shape (horizontally elongated), irregular in size, preovarian, arranged in two fields, each with a single row of testes. Testes 23–43 (34 ± 9, n = 8) long and 66–109 (89 ± 14, n = 8) wide (Figures 2A, 5A, 5C); length-width ratio L:W = 1: 1.8. Vas deferens dorsal to testes, goes in the direction of the anterior region; in the part anterior to the testicular region, widest in region between testicular region and cecal bifurcation, coiled, anterior to cecal bifurcation, narrow and straight to connection with MCO (Figures 2A, 5A). Male copulatory organ muscular, subspherical, 37–52 (45 ± 6, n = 8) long, 40–50 (44 ± 4, n = 8) wide, armed with 18–26 (24 ± 2, n = 8) spines (Figures 2B, 5A, 5B, 6A, 6B). Spines of MCO hooklike, 25–31 (28 ± 2, n = 8) long, 2–4 (3 ± 1, n = 8) wide, arranged in a circle with tips directed outward (Figures 2B, 2C, 6A, 6B). Genital atrium ventral, near midline, anterior to cecal bifurcation, opening 190–350 (257 ± 48, n = 8) from anterior end of body (Figures 2A, 5A, 5B, 6A), subspherical, with muscular edges and without any sclerotized structures.

*Female reproductive structures* (Figures 2A, 2D, 4, 6A, 6B). Germarium intercecal, post-testicular, comprised of oval germarial bulb with irregular edges, containing immature oocytes, 48–104 (74 ± 20, n = 6) long, 60–103 (80 ± 15, n = 6) wide (Figure 4A). Ascending duct reaches anteriorly past vitelline duct, bent slightly to the right (Figure 5A) or left (Figures 2A, 4A) side, forming an inverted U that crosses the ascending duct to descend towards the oötype (Figures 2A, 4A, 5A). Vaginal duct on left side, ventral to common vitelline duct, seminal receptacle dorsal to common vitelline duct; ducts connect to oötype. Genito-intestinal canal observed (Figure 4A). Uterus ascends from oötype to the genital atrium (Figure 4A). Vaginal pore ventral, anterior to vaginal vestibule. Vaginal vestibule 54–88 (75 ± 10, n = 8) long, 44–58 (51 ± 5, n = 8) wide (Figures 2A, 2B, 5A, 5B, 6A, 6B), located 270–445 (346 ± 56, n = 8) from anterior end of body and 70–180 (120 ± 24, n = 8) from MCO. Vaginal vestibule lateral to midline, on the side opposite to haptoral clamps (Figures 2A, 2D, 5A, 5B, 6A, 6B). Vaginal vestibule armed with 43–58 (52 ± 4, n = 6) spines. Spines 23–26 (25 ± 1, n = 8) long, 2–4 (2, n = 14) wide (Figure 2E). Spines flattened, variable in length with basal spines smallest, uppermost spines largest. Each spine with small needle-like spikes at the distal end (Figures 2A, 2D, 2E, 5A, 5B, 6A, 6B). Vaginal duct descends from vaginal vestibule to germarium; small elongate seminal receptacle dorsal to common vitelline duct, observed in some specimens, not measured (Figure 4A). Vitelline glands in two lateral fields, starting just posterior to cecal bifurcation, overlapping ceca anteriorly and posteriorly, reaching lateral margin of testes, uniting posterior to germarium, extending to posterior region of body but not into haptoral lappet (Figure 2A). Vitellogenic ducts unite dorsal to anterior part of germarium, to form common vitelline duct that is connected to oötype (Figures 4A, 5A).

*Eggs* (Figure 4B). Some specimens with single egg in uterus. Eggs eliptoid, with two polar filaments; length, not including polar filaments, 136–166 (155 ± 9, n = 4) long, 42–53 (48 ± 5, n = 4) wide. Anterior filament (as positioned in uterus) 209 (n = 1) long by 5 (n = 1) wide; posterior filament 260 (n = 1) long by 6 (n = 1) wide.

### Taxonomic summary

**Type Host:***Caranx hippos* (Linnaeus, 1766) (Carangidae).

**Common name:** Crevalle jack.

**Site of infection:** Gills filaments.

**Type locality:** Littoral waters of the Gulf of Mexico, off Tecolutla, Veracruz (20°28′39″N; 97°00′30″W) (Figure 1).

**Specimens deposited:** Holotype CNHE 12575; Paratypes CNHE 12576; HWML 218464–218466; CHE-P00155.

**ZooBank registration:** BB2B1819-B0E0-4FBD-BBE3-D59BB85FEBFF.

**Genbank accession numbers:** mtDNA: PZ380651 to PZ380654. 28S rDNA: PZ377455–PZ377458.

**Etymology:** The species is named in honor of Dra. Elizabeth A. Martínez-Salazar (Unidad Académica de Ciencias Biológicas, Universidad Autónoma de Zacatecas, Zacatecas, Mexico) for her contributions to the academic training of the first author, her contributions to the fields of Helminthology and Molecular Systematics, and her friendship.

### Remarks

Morphological characteristics, including the number of testes, number and shape of the spines on the male copulatory organ, and the number of vaginal spines (Bravo-Hollis, 1966; Kritsky et al., 2011; Pillai & Pillai, 1978; Ramalingam, 1960; Wahl, 1972; Yamaguti, 1953), as well as the body size and shape, and the arrangement and shape of the testes (Kritsky et al., 2011; Ramírez-Cruz et al., 2023), have been employed as diagnostic characters for the differentiation of species of *Protomicrocotyle*. These and other characters indicate that the new species should be assigned to *Protomicrocotyle* and are useful for distinguishing the new species from other known members of the genus.

The testes of *P. eamsae* sp. nov. are arranged in two fields, as in *P*.*mirabilis* (Kritsky et al., 2011; Ramírez-Cruz et al., 2023), *P*. *carangis* (Pillai & Pillai, 1978) and *P*.*mannarensis* (Ramalingam, 1960); The testes of *P*. *veracruzensis*, *P*. *minutum*, and *P*.*nayaritensis* are arranged in rows with 1–2 adjacent testes on each side (Bravo-Hollis, 1966; Ramalingam, 1960), in *P*. *madrasensis* they appear to be in row with 2 adjacent testes on each side (Ramalingam, 1960), and those of *P*. *ivoriensis* have rows with 1–3 adjacent testes (Wahl, 1972). In the new species, the testes are shallowly doliform (horizontally elongated) (Figures 2A, 5A, 5C) and in *P*.*mirabilis*, the testes are very broadly eliptoid (Kritsky et al., 2011; Ramírez-Cruz et al., 2023). Furthermore, the new species has fewer testes (31, in two fields) than *P*.*nayaritensis* (46–48, in a single field) (Bravo-Hollis, 1979).

The body of *P. eamsae* sp. nov. is shorter in length (1682) than most other species of *Protomicrocotyle* reported from the coasts the Eastern Pacific Ocean and Gulf of Mexico (Table 3); the only species that is similar in size is *P*.*minutum* (1500), from India (Ramalingam, 1960).

*Protomicrocotyle eamsae* sp. nov. has fewer spines on the MCO and more spines on the vaginal vestibule than *P. manteri* (24 vs. 33–38 and 52 vs. 15, respectively) (Bravo-Hollis, 1966) (Table 3). The new species and *P*.*nayaritensis* are similar to *P. manteri* in having an average of 24 spines in the MCO and is different from *P*.*nayaritensis*, which has 48–54 spines in the MCO. Additionally, the new species, with 52 spines in the vaginal vestibule, can be distinguished from *P*.*nayaritensis*, which has 22–46 spines in the vaginal vestibule.

The testes of each field of *P. eamsae* sp. nov. are arranged in a single row, while in *P*.*veracruzensis,* the testes of each field occasionally have one or two smaller-sized testes interspersed between the larger testes to form an irregular column, rather than having all in a single row (Ramírez-Cruz et al., 2023). As well, the new species is shorter in body length than *P*.*veracruzensis* (1682 vs. 3858, respectively) and the new species has 31 testes and *P*.*veracruzensis* has 47 (Table 3).

Finally, this new species can be distinguished from *P. mirabilis*, *P*.*veracruzensis*, *P*.*nayaritensis* and the second new species described below by the presence of the retractile glandulomuscular organ in the prohaptor, which has only been documented in *P*.*manteri* from the Pacific Ocean (Bravo-Hollis, 1966). Additionally, it can be distinguished by the slightly lobed lateral ends of the haptoral lappet (Figures 2A, 5A, 5D).

### *Protomicrocotylepritchardae* sp. nov.

**Figure 7:**
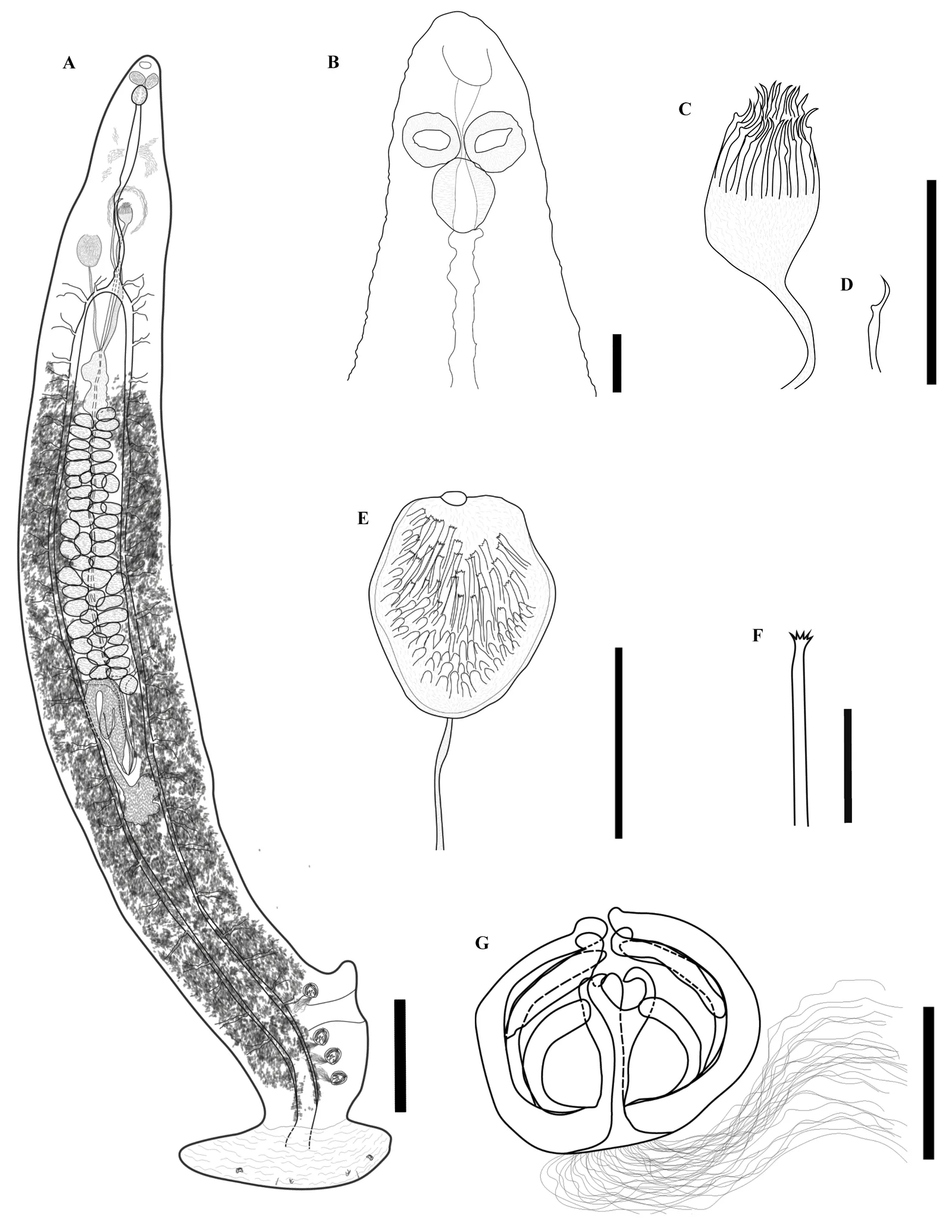
Line drawings of the holotype (CNHE 12577) of *Protomicrocotyle pritchardae* sp. nov. from the gills of *Caranx hippos*. (A) Complete body, ventral view. Bar 100 μm; (B) Anterior region. Bar 50 μm; (C) Masculine copulatory organ. Bar 100 μm; (D) Spine of MCO. Bar 100 μm; (E) Vaginal vestibule. Bar 100 μm; (F) Spine of vaginal vestibule. Bar 25 μm; (G) Clamp with muscular peduncle. Bar 32 μm.

*Description* (Figures 7 [Fig gf08] [Fig gf09]–10; Tables 2, 3)*.* Based on 11 specimens, stained, and mounted to be viewed from ventral side of the body. Some comparative measurements and data of other species are presented in Tables 2 and 3.

**Figure 8 gf08:**
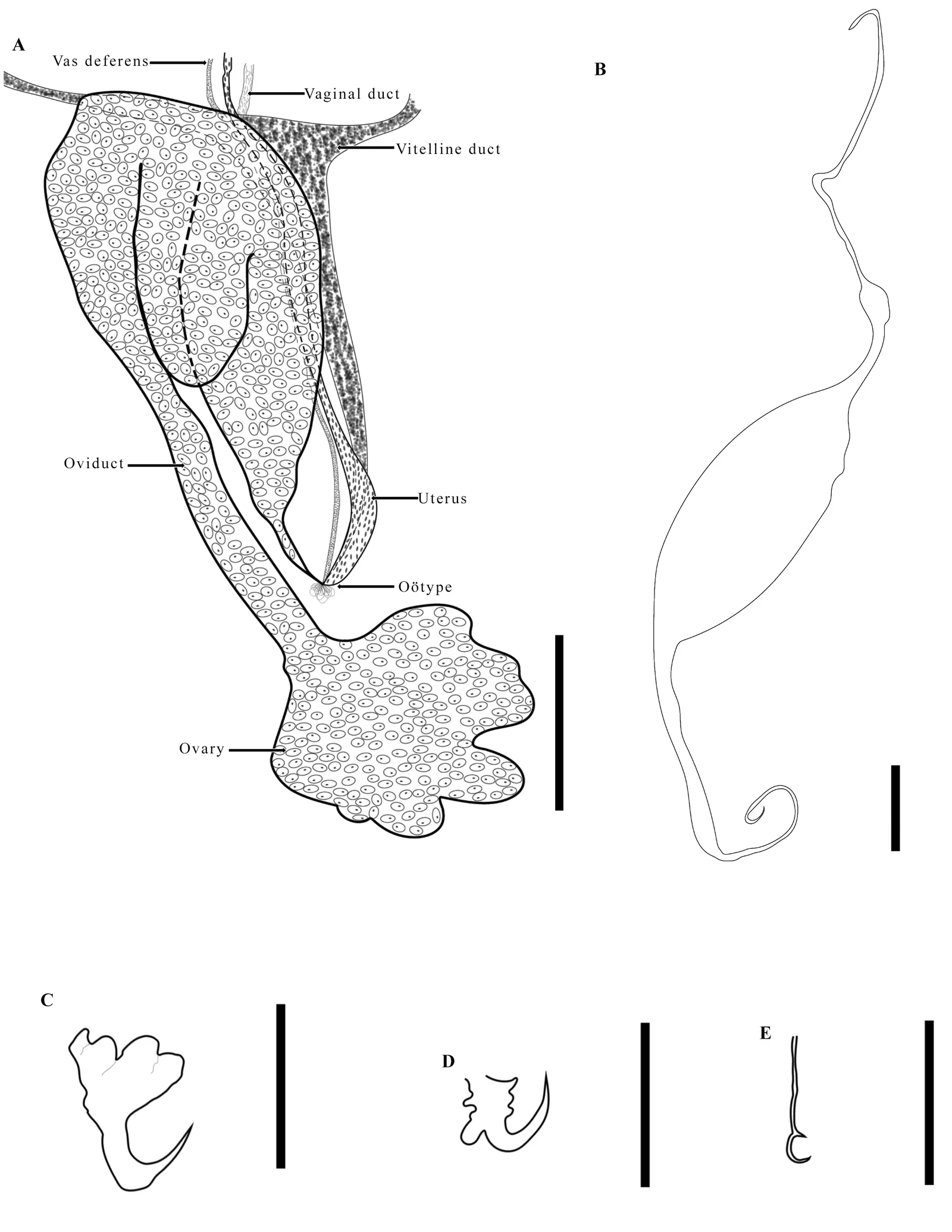
(A) Reproductive organs of *Protomicrocotyle pritchardae* sp. nov. (CNHE 12577). Bar 100 μm; (B) Egg with two long polar filaments (paratype CNHE 12578). Bar 50 μm; (C) Lateral anchor (CNHE 12578). Bar 16 μm; (D) Median anchor (CNHE 12578). Bar 16 μm; (E) Hook (CNHE 12578). Bar 16 μm.

**Figure 9 gf09:**
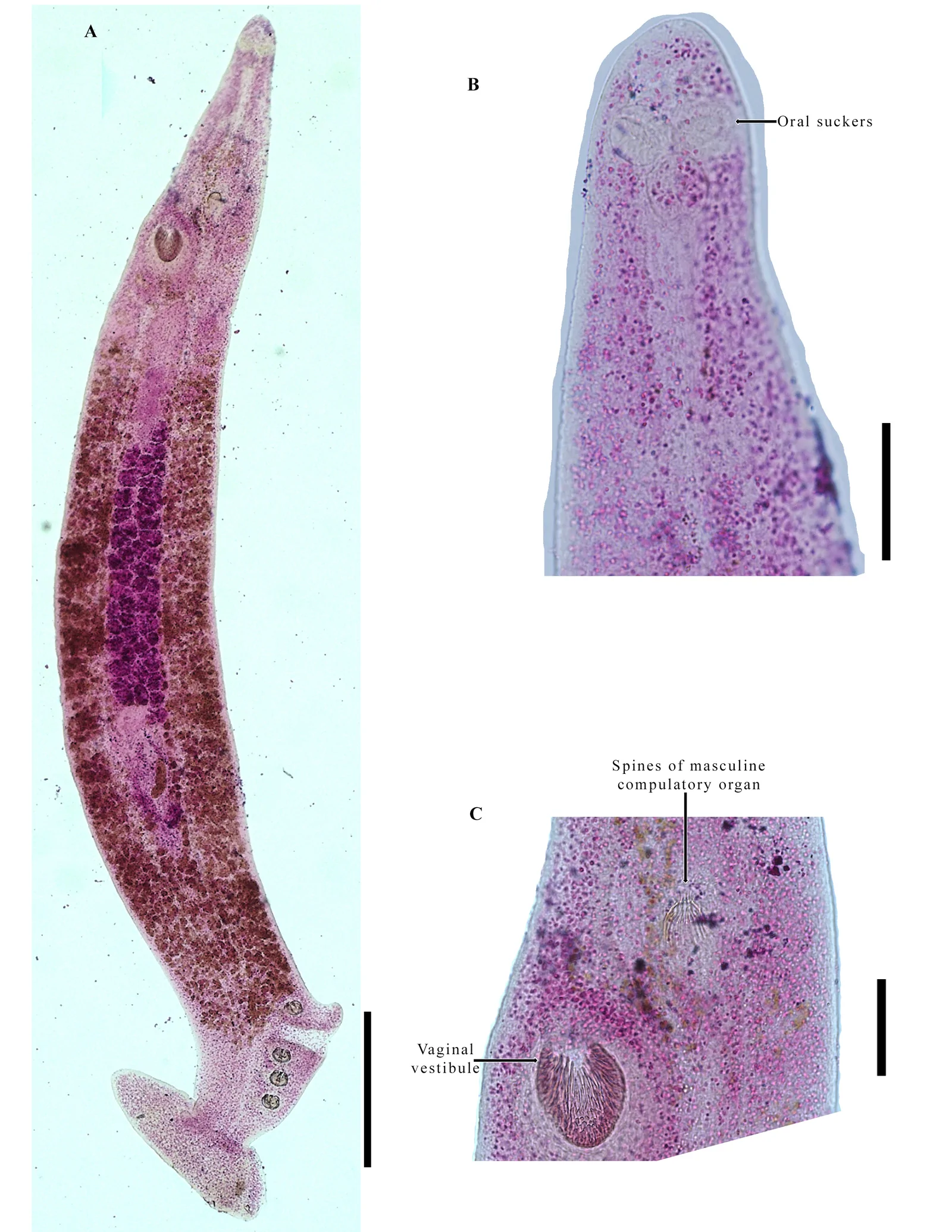
Light micrographs of the paratype (CNHE 12578) of *Protomicrocotyle pritchardae* sp. nov. (A) complete body, ventral view. Bar 500 μm; (B) Prohaptor. Bar 100 μm; (C) Details of the masculine copulatory organ and vestibule vaginal. Bar 100 μm. Delafield’s hematoxylin stain.

**Figure 10:**
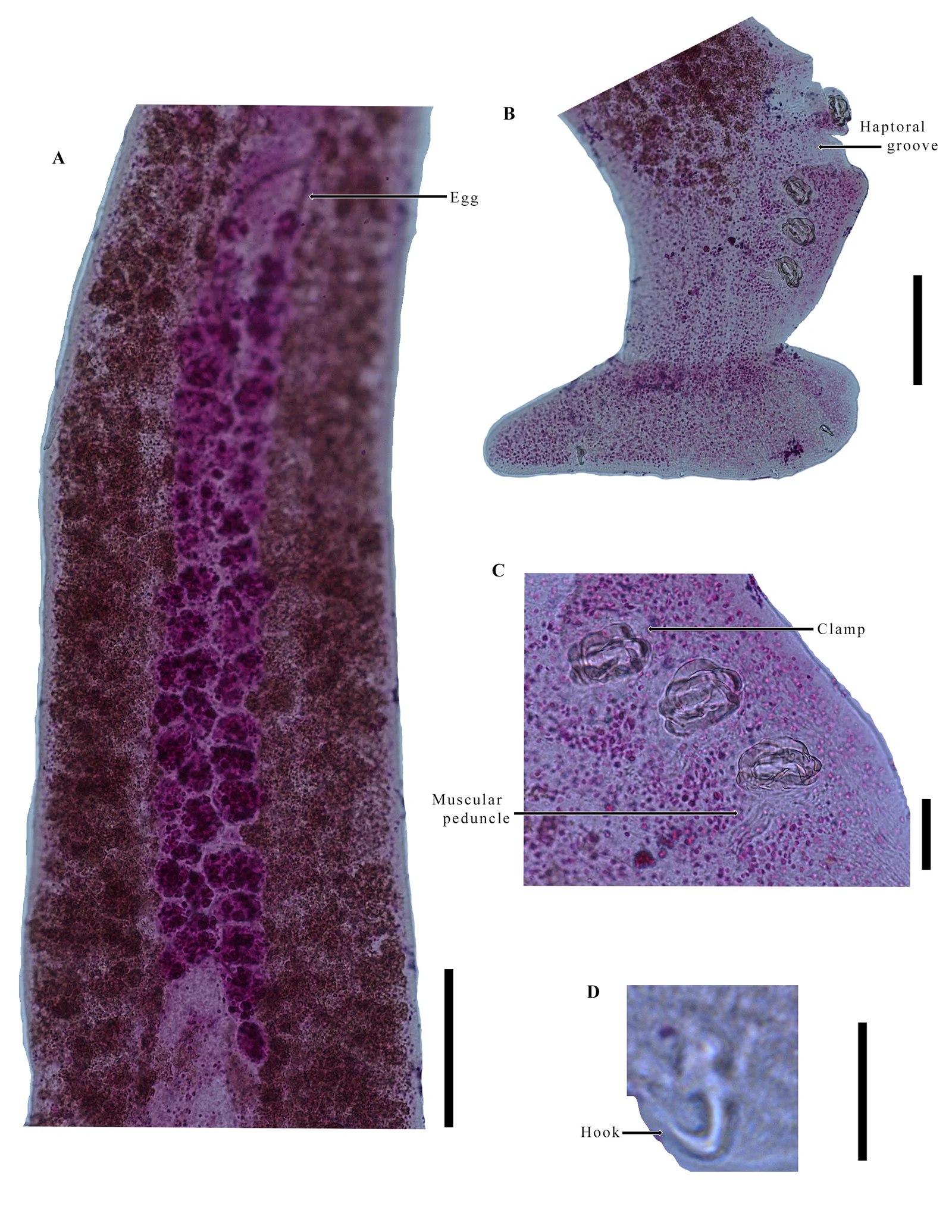
Light micrographs of the paratype (CNHE 12578) of *Protomicrocotyle pritchardae* sp. nov. (A) Body trunk. Bar 200 μm; (B) Haptor. Bar 200 μm; (C) Clamps. Bar 50 μm; (D) Median anchor. Bar 20 μm.

*Body* (Figures 7A, 9A). Body fusiform; anterior end conical. Body length 3721–4380 (4117 ± 246, n = 11) including the haptor; width 488–573 (537 ± 27, n = 11) (Figures 7A, 9A). Tegument with light cuticular striations, mainly in the middle and posterior regions of the body. Paired prohaptoral suckers in the anterior region, septate, muscular, oblique, and very shallowly dolliform; right pseudo-sucker 42–59 (52 ± 5, n = 11) long, 40–48 (43 ± 2, n = 11) wide; left pseudo-sucker 47–60 (54 ± 5, n = 11) long, 42–55 (44 ± 2, n = 11) wide (Figures 7B, 9B). Aperture oral ventral, subterminal, finely ovoid, 31–35 (34 ± 1, n = 3) long, 25–59 (42 ± 10, n = 8) wide (Figures 7B, 9B). Glandomuscular organ absent. Pre-pharynx absent; pharynx broadly elliptoid, muscular, 30–55 (45 ± 6, n = 14) long, 40–48 (43 ± 3, n = 14) wide (Figures 7B, 9B). Esophagus 605–765 (663 ± 46, n = 10) long, 20–30 (24 ± 3, n = 10) wide, with diverticula (Figures 7A, 9A, 10A). Cecal bifurcation posterior to MCO, 775–920 (846 ± 51, n = 10) from anterior end (Figures 7A, 9A). Ceca lateral to midline, extending posteriorly to haptoral region but failing to reach haptoral lappet; cecal diverticula present that extend to margins of body (Figures 7A, 9A).

*Haptor* (Figures 7A, 9A, 10B, 10C, 10D)*.* Haptor asymmetrical, armed with a row of four gastrocotylid-type clamps, each with a short muscular peduncle; haptoral groove between first and second clamps (Figures 7A, 7G, 9A, 10B). Haptor 655–835 (726 ± 52, n = 10) long, 410–470 (438 ± 19, n = 10) wide at the level of second clamp (Figures 9A, 10B). Clamps are similar in size: first clamp 36–47 (41 ± 3, n = 8) long by 49–58 (54 ± 3, n = 8) wide; second clamp 42–50 (46 ± 3, n = 8) long by 54–62 (41± 5, n = 10) wide; third clamp 41–55 (46 ± 4, n = 10) long by 54–65 (59 ± 3, n = 10) wide; and, fourth clamp 40–56 (48 ± 6, n = 9) long by 54–62 (59 ± 3, n = 8) wide (Figure 7G, 10C). Haptoral lappet transversely elongated (Figures 7A, 10B); right lateral length 145–187 (174 ± 12, n = 10), left lateral length 124–198 (169 ± 21, n = 10), central length 170–220 (196 ± 14, n = 10). Haptoral lappet 645–820 (726 ± 48, n = 11) wide, armed with two pairs of anchors and one pair of hooks (Figures 8CE, -10 D; Table 2).

*Male reproductive structures* (Figures 7A, 7CD, -9A, 9 C, 10A). Testes intercecal, 53–67 (60 ± 5, n = 11) in number, broadly elliptoid (subspherical), irregular in size, pre-ovarian, arranged in a single field (Figures 7A, 9A, 10A). Testes 43–66 (53 ± 8, n = 11) long and 65–102 (82 ± 14, n = 11) wide (Figures 7A, 9A, 10A); length-width ratio L:W = 1:1.5. Vas deferens dorsal to testes, goes in the direction of the anterior region; in the part anterior to the testicular region, widest in region between testicular region and cecal bifurcation, coiled, posterior to cecal bifurcation, narrow and straight to connection with MCO (Figures 7A, 9A). Male copulatory organ muscular, subspherical, 59–74 (64 ± 4, n = 10) long, 43–55 (49 ± 3, n = 11) wide, armed with 13–35 (21 ± 7, n = 11) spines. Spines of MCO hooklike, 34–49 (42 ± 4, n = 11) long, 2–5 (4 ± 1, n = 11) wide, arranged in a circle on anterior part of MCO (Figures 7A, 7C, 9A, 9C). Each spine with small knob in the anterior region just posterior to curved tip, knob and curved tip directed outwards (Figures 7C, 7D). Genital atrium ventral, near midline, anterior to cecal bifurcation, opening 500–625 (552 ± 40, n = 11) from anterior end of body, subspherical, with muscular edges and without sclerotized structures (Figures 7A, 9A).

*Female reproductive structures* (Figures 7A, 7E, 8A, 8B, 9A, 9C). Germarium intercecal, post-testicular, comprised of germarial bulb with lobed edges, containing immature oocytes, 124–193 (157 ± 21, n = 9) long, 72–131 (102 ± 16, n = 9) wide (Figures 7A, 7E, 8A). The ascending duct extends towards the anterior region and turns to the right side forming an inverted U, then descends towards the oötype. Vaginal duct and vitelline duct connect to oötype; uterus ascends from oötype to the genital atrium (Figure 8A). Seminal receptacle and genito-intestinal canal not observed. Vaginal pore ventral, anterior to the vaginal vestibule. Vaginal vestibule 101–133 (113 ± 9, n = 11) long, 76–92 (84 ± 6, n = 11) wide (Figures 7A, 7E, 9A, 9C), located 635–755 (690 ± 46, n = 11) from anterior end of body and 150–210 (179 ± 17, n = 11) from the genital pore, lateral to midline on the side opposite to that having the haptoral clamps. Vaginal vestibule armed with 35–61 (45 ± 7, n = 11) spines. Spines 34–47 (41 ± 3, n = 11) long, 4–6 (5 ± 1, n = 11) wide; flat shape, variable in size. Basal spines smallest, uppermost spines largest. Each spine elongated in shape with small needle-like spikes at the distal end (Figure 7F). Vaginal duct descends from vaginal vestibule to germarium, simple, without seminal receptacle (Figure 7A, 8A). Vitelline glands in two lateral fields, starting just posterior to cecal bifurcation, overlapping ceca anteriorly and posteriorly and the lateral margin of the testes, uniting posterior to germarium, extending to posterior region of body but not reaching the haptoral lappet; in some specimens, clusters of glands are observed at the level of the cecal bifurcation and vaginal vestibule (Figures 7A, 9A, 9C). Vitellogenic ducts from each field unite dorsal to the ducts of the germarium, to forming common vitelline duct that connects to the oötype (Figure 8A).

*Eggs* (Figures 8B, 10A). Some specimens with single egg in uterus (n = 9). Eggs elliptoid, with two polar filaments, at each pole; length, not including polar filaments, 164–280 (192 ± 21, n = 9) long, 72–131 (102 ± 16, n = 9) wide. Filament in anterior end (as positioned in uterus) 125–244 (193 ± 40, n = 7) long by 6–7 (7, n = 7) wide; posterior filament 268–325 (296 ± 41, n = 2) long by 5–7 (6 ± 1, n = 2) wide (Figure 8B).

### Taxonomic summary

**Type Host:***Caranx hippos* (Linnaeus, 1766) (Carangidae).

**Common name:** Crevalle jack.

**Site of infection:** Gills filaments.

**Type locality:** Littoral waters of the Gulf of Mexico off Tecolutla, Veracruz (20° 28′ 39″ N; 97° 00′ 30″ W) (Figure 1).

**Specimens deposited:** Holotype CNHE 12577; Paratypes CNHE 12578; HWML 218467–218470; CHE-P00154.

**ZooBank registration:** BB2B1819-B0E0-4FBD-BBE3-D59BB85FEBFF.

**Genbank accession numbers:** mtDNA: PZ380648–PZ380650. 28S rDNA: PZ377459–PZ377462.

**Etymology:** The species is named in honor of Dra. Mary Louise Hanson Pritchard, founding curator of the H.W. Manter Laboratory, Division of Parasitology at the State Museum of the Nebraska University, for her contributions to parasitology and the HWML Museum.

### Remarks

The diagnostic characters mentioned above, and other characters indicate that this new species should be assigned to *Protomicrocotyle* and are useful for distinguishing the new species from other known members of the genus.

*Protomicrocotyle pritchardae* sp. nov. differs from *P*.*mirabilis* by being longer, with a mean length of 4117 vs. 2861, respectively (Table 3). The shape of the testes is similar in both species (broadly elliptoid to very broadly elliptoid), the new species has a greater number of testes (60 vs. 31, respectively). The testes are arranged in a single field in the new species, whereas in *P*.*mirabilis* the testes are in two well-defined rows of testes (Kritsky et al., 2011; Ramírez-Cruz et al., 2023). The new species has less spines on the MCO compared to *P*.*manteri* (21 vs. 33–38) and more spines within the vaginal vestibule (52 vs. 15, respectively) (Table 3). The new species has broadly elliptoid testes, whereas *P*.*manteri* has depressed elliptoid testes (Bravo-Hollis, 1966). The new species has less spines in the MCO than *P*.*nayaritensis* (21 vs 48–54, respectively) (Table 3); the new species has 35–61 spines in the vaginal vestibule and *P*.*nayaritensis* has 22–46 (Bravo-Hollis, 1979). The new species and *P*.*veracruzensis* are similar in body length (Table 3); however, they can be differentiated by the size of the clamps (45 in the new species vs. 64 in *P*.*veracruzensis*) and the average number of testes (60 in the new species vs. 47 in *P*.*veracruzensis*). In addition, the new species has testes that are broadly elliptoid and arranged in a single field, and *P*.*veracruzensis* has testes that are mostly depressed elliptoid in shape and they are arranged in two parallel fields, each field with one or two interspersed testes, forming an irregular column (Ramírez-Cruz et al., 2023).

Finally, *P. mirabilis*, *P*.*eamsae* sp. nov., and *P*.*pritchardae* sp. nov. are found in sympatry as parasites of *C*. *hippos* in the same geographic area of Tecolutla, on the southwestern coast of the Gulf of Mexico. It is noteworthy that, among the fish that were examined, *P*.*mirabilis* and *P*.*eamsae* sp. nov. sometimes co-infected the same fish; whereas, *P*.*pritchardae* sp. nov. was present as a single-species infection in only three fish, without co-infection with other species *of Protomicrocotyle*. Whether this patter is because of the relatively small sample size of fish, or is indicative of possible separation of populations of fish is unknown.

### Morphometric analyses

**Figure 11:**
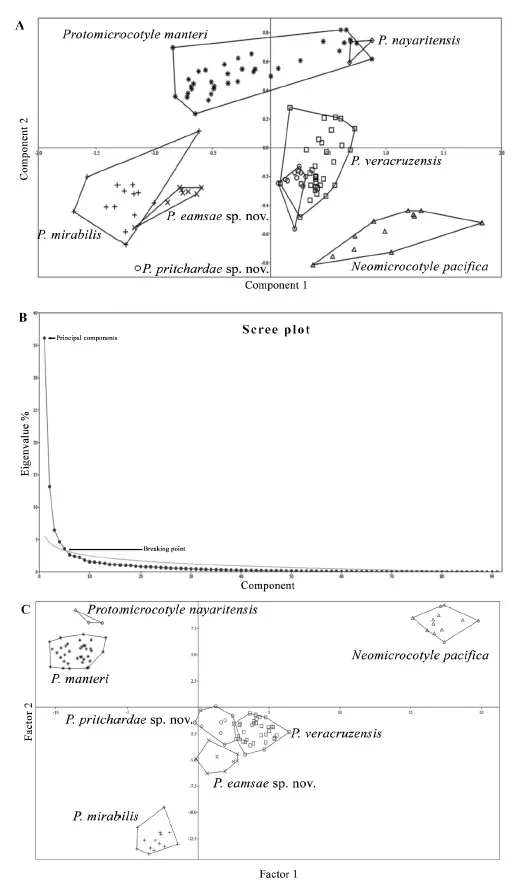
Morphometric analyses of the species of *Protomicrocotyle*. (A) Scree plot of the PCA; (B) Scatter plot of PCA; (C) Scatter plot of LDA.

Based on the scree plot (Figure11A), the first two components explained 86.28% of the accumulated variance (Figures 11A, 11B; Table 4A). The curve of the steep slope stabilizes and flattened out after components three and four so they were not considered. (Figure 11A). In order of importance, the morphological variables that contributed to the first component were: the total width of the body, the width of the ovary, the width of the haptor, the distance from vaginal vestibule to genital pore, the number of testes, the right lateral length of the haptoral lappet, left lateral length of haptoral lappet, central length of the haptoral lappet, the width of haptoral lappet, and the total body length (Table 5). For the second component, the morphological variables were: the number of spines on the male copulatory organ, the average width of the testes, the length of the external root of the right lateral hook, the length of the opening of the left median hook, the width and length of the ovary (Table 5).

**Table 4:** The cumulative variance of the first two principal components (**A**) and the factor values of the linear discriminant analyses (**B**).

**A**			
PC	Eigenvalue	% Variance	% Cumulative variance
1	0.894714	62.93	62.93
2	0.33193	23.34	86.28
**B**			
Factor	Eigenvalue	% Variance	% Cumulative variance
1	64.297	48.71	48.71
2	41.643	31.55	80.26

**Table 5:** Loadings derived from PCA of *Protomicrocotyle* morphological variables.

**Morphological variables**	**Principal component 1**	**Morphological variables**	**Principal component 2**
Body width	0.29285	Number of spines in the male copulatory organ	0.33702
Ovary width	0.23174	Average testes width	0.17884
Haptor width	0.22967	External root length of the right lateral hook	0.15014
Distance from vaginal vestibule to genital pore	0.21108	Opening length of the left median hook	0.14368
Testes number	0.20845	Ovary width	0.14183
Right lateral length of the haptoral lappet	0.20503	Ovary length	0.14105
Left lateral length of the lappet	0.19834	Internal root width of the right middle hook	0.13752
Central length of the haptoral lappet	0.19293	Deep root length of the left medial anchor	0.12418
Male copulatory organ length	0.19064	Vaginal spine width	0.11695
Haptoral lappet width	0.18955	Internal root width of the right lateral anchor	0.10917
Body length	0.18687	Root Left outer anchor superficial root length	0.10834
Male copulatory organ spines length	0.18076	Esophagus width	0.10407
Haptor length	0.17259	External root length of the left lateral anchor	0.10351
Average testes width	0.16943	Haptor width	0.096796
Distance from genital pore to anterior end	0.1645	Length of the right lateral anchor	0.093181
Number of spines in the vaginal vestibule	0.15855	Point length of the left lateral anchor	0.091329
Distance from vaginal vestibule to anterior end	0.14964	Body width	0.089647
Ovary length	0.13922	Internal root length of the right medial anchor	0.088829
Vaginal vestibule width	0.13326	Hook width of the left central hook	0.08715
Oral aperture width	0.13233	Distance from vaginal vestibule to genital pore	0.086178
Third clamp width	0.12297	Anchor width of the right medial anchor	0.08453
First clamp width	0.12274	Internal root length of the right medial anchor	0.083183
Left pseudosucker length	0.12264	Male copulatory organ width	0.08206
Male copulatory organ width	0.12235	Opening length of the right lateral anchor	0.076877
Second clamp width	0.12234	Right pseudosucker length	0.073615
Right pseudosucker length	0.1182	Opening length of the right medial anchor	0.073491
Pharynx length	0.11167	Internal root length of the left medial anchor	0.069338
Fourth clamp width	0.10982	Distance from vaginal vestibule to anterior end	0.067462
Pharynx width	0.10559	Left pseudosucker length	0.066529
Vaginal vestibule length	0.10554	Body length	0.063611
Average testes length	0.10397	Point length of the right lateral anchor	0.06256
Male copulatory organ spines width	0.098505	Left outer anchor length	0.061505
Left pseudosucker width	0.098178	Internal root width of the left lateral anchor	0.059995
Right pseudosucker width	0.09791	Internal root length of the right lateral anchor	0.058184
Vaginal spines width	0.093731	Average testes length	0.057786
Opening length of the left inner anchor	0.087836	Hook width of the right central hook	0.046617
External root length of the right lateral anchor	0.085656	Main portion length of the left lateral anchor	0.045046
Second clamp length	0.076151	Distance from genital pore to anterior end	0.044954
Third clamp length	0.075501	Opening width of the left lateral anchor	0.044657
Internal root length of the right lateral anchor	0.075305	Main portion length of the right lateral anchor	0.0423
Oral aperture length	0.072972	Handle length of the right lateral hook	0.038723
Esophagus length	0.072496	Width of the left middle hook	0.037119
Internal root width of the right lateral anchor	0.068715	Total length of the left central hook	0.035172
Distance from cecal bifurcation to anterior end	0.065186	Vaginal spines length	0.034826
Fourth clamp length	0.060103	Width of the right lateral anchor	0.033039
First clamp length	0.058903	Central length of the haptoral lappet	0.031987
Esophagus width	0.055491	Main point length of the left lateral anchor	0.031082
Opening length of the right lateral hook	0.052178	Haptor length	0.026203
Length of the right lateral anchor	0.048302	Total length of the right central hook	0.02508
Internal root width of the left lateral anchor	0.047862	Oral aperture width	0.023876
Width of the left middle hook	0.042895	Oral aperture length	0.023023
Internal root width of the right middle hook	0.038124	Main point length of the right median anchor	0.022088
Width of the right middle hook	0.035793	Anterior filament length	0.019909
Length of the left lateral anchor	0.034459	Esophagus length	0.014519
Handle length of the right lateral hook	0.034307	Main point length of the right median anchor	0.01082

The accumulated variance demonstrated by the LDA was 80.26% (Table 4B). Seven groups were supported in the scatter plot (Figure 11C), six of which corresponded to species of *Protomicrocotyle* and one to *Neomicrocotyle pacifica*. The four species of *Protomicrocotyle* from the Gulf of Mexico (*P*. *mirabilis*, *P*. *veracruzensis*, *P*. *eamsae* sp. nov., and *P*. *pritchardae* sp. nov.) were more similar to each other than to those species from the Pacific Ocean: *P*. *manteri*, *P*.*nayaritensis*, and *N*. *pacifica* (Figure 11C). The specimens of *Protomicrocotyle pritchardae* sp. nov. and *P*. *veracruzensis* share some similar morphological characteristics (longitude of the esophagus, distance from the cecal bifurcation to the anterior end) so they are not completely separated (Figure 11C).

The original assignment of specimens to species was supported 100% CC by the original resubstituting matrix assignment of the LDA (Table 6A), but application of the cross-validation reduced the percentage of correctly assignment to 95.73% CC (Table 6B). The eight specimens of *P*. *eamsae* sp. nov. were assigned correctly; although, one of 11 specimens of *P*. *pritchardae* sp. nov. was grouped with *P. veracruzensis* (Table 6B). However, based on all of the analyses, the morphological comparison of the specimens, and molecular data, the specimen is correctly assigned to *P*. *pritchardae* sp. nov.

**Table 6:** Confusion matrices for the linear discriminant analyses. (**A**) Original resubstituting matrix (100% CC). (**B**) Jackknife matrix (*Cross-Validation*) (95.73% CC).

**A**								
**Species**	***Protomicrocotyle mirabilis***	***P. veracruzensis***	***P. eamsae***	***P. pritchardae***	***P. nayaritensis***	***P. manteri***	***Neomicrocotyle pacifica***	**Total**
*Protomicrocotyle mirabilis*	14	0	0	0	0	0	0	14
*P. veracruzensis*	0	33	0	0	0	0	0	33
*P. eamsae*	0	0	8	0	0	0	0	8
*P. pritchardae*	0	0	0	11	0	0	0	11
*P. nayaritensis*	0	0	0	0	3	0	0	3
*P. manteri*	0	0	0	0	0	36	0	36
*Neomicrocotyle pacifica*	0	0	0	0	0	0	12	12
Total	14	33	8	11	3	36	12	117
**B**								
**Species**	***Protomicrocotyle mirabilis***	***P. veracruzensis***	***P. eamsae***	***P. pritchardae***	***P. nayaritensis***	***P. manteri***	***Neomicrocotyle pacifica***	**Total**
*Protomicrocotyle mirabilis*	14	0	0	0	0	0	0	14
*P. veracruzensis*	0	29	0	4	0	0	0	33
*P. eamsae*	0	0	8	0	0	0	0	8
*P. pritchardae*	0	1	0	10	0	0	0	11
*P. nayaritensis*	0	0	0	0	3	0	0	3
*P. manteri*	0	0	0	0	0	36	0	36
*Neomicrocotyle pacifica*	0	0	0	0	0	0	12	12
Total	14	30	8	14	3	36	12	117

### Molecular analyses

Seven partial sequences of CO1 mtDNA were obtained from the new species (four from *P. eamsae* sp. nov. and three from *P. pritchardae* sp. nov.) and eight partial sequences of 28S rDNA (four from *P. eamsae* sp. nov. and four from *P. pritchardae* sp. nov.). The newly obtained partial sequences and those retrieved from GenBank® (Sayers et al., 2020) for each gene (Table 1) were aligned; the final alignment of CO1 mtDNA was the 393 base pair (bp) and that of 28S rDNA was 760 bp. The intraspecific genetic variation of CO1 mtDNA and 28S rDNA are given in Tables 66B.

**Figure 12:**
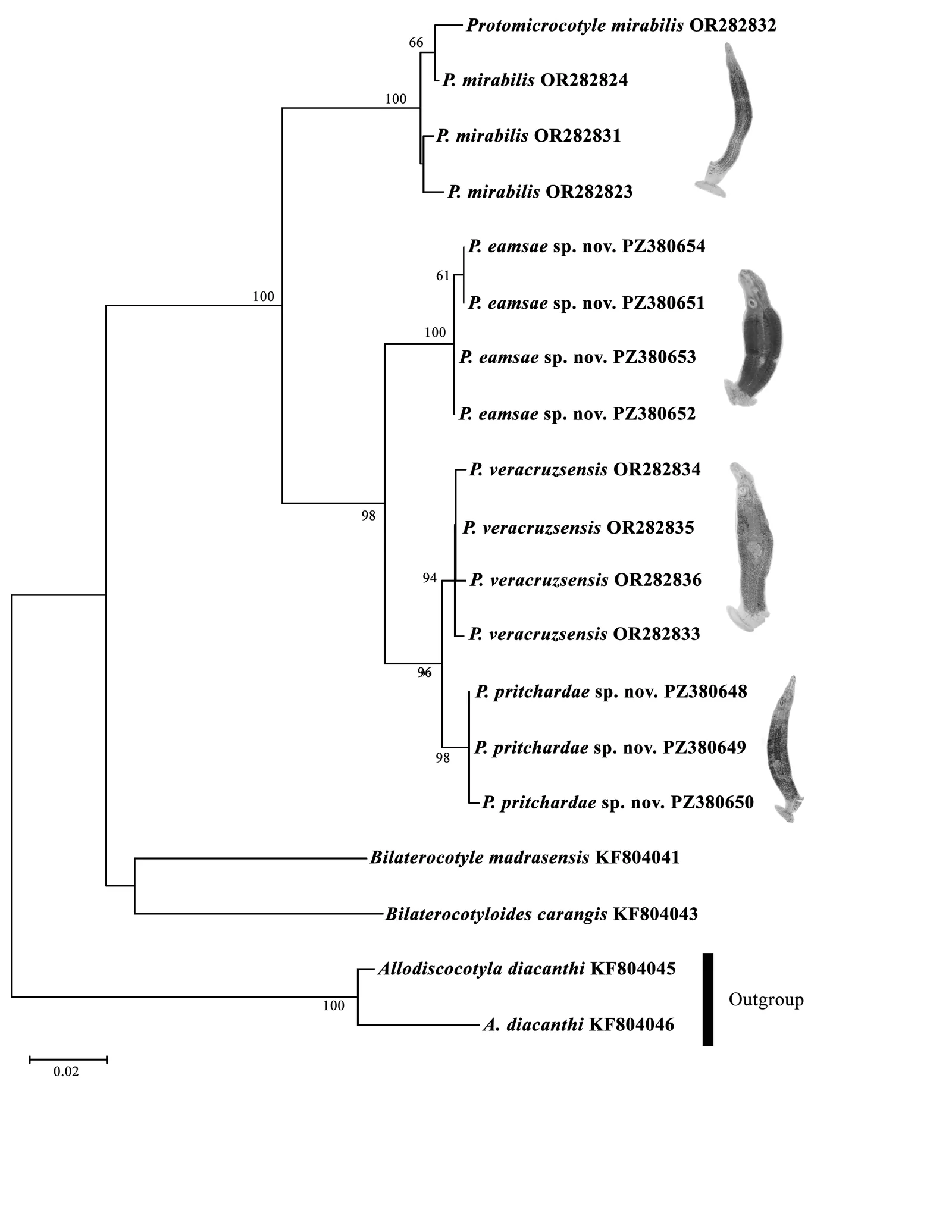
Neighbor Joining phenogram based on CO1 mtDNA gene sequences showing similarities between new species of *Protomicrocotyle* and other protomicrocotylids. Bootstrap support values calculated for 1000 replications are indicated. Accession numbers from GenBank are given.

**Figure 13:**
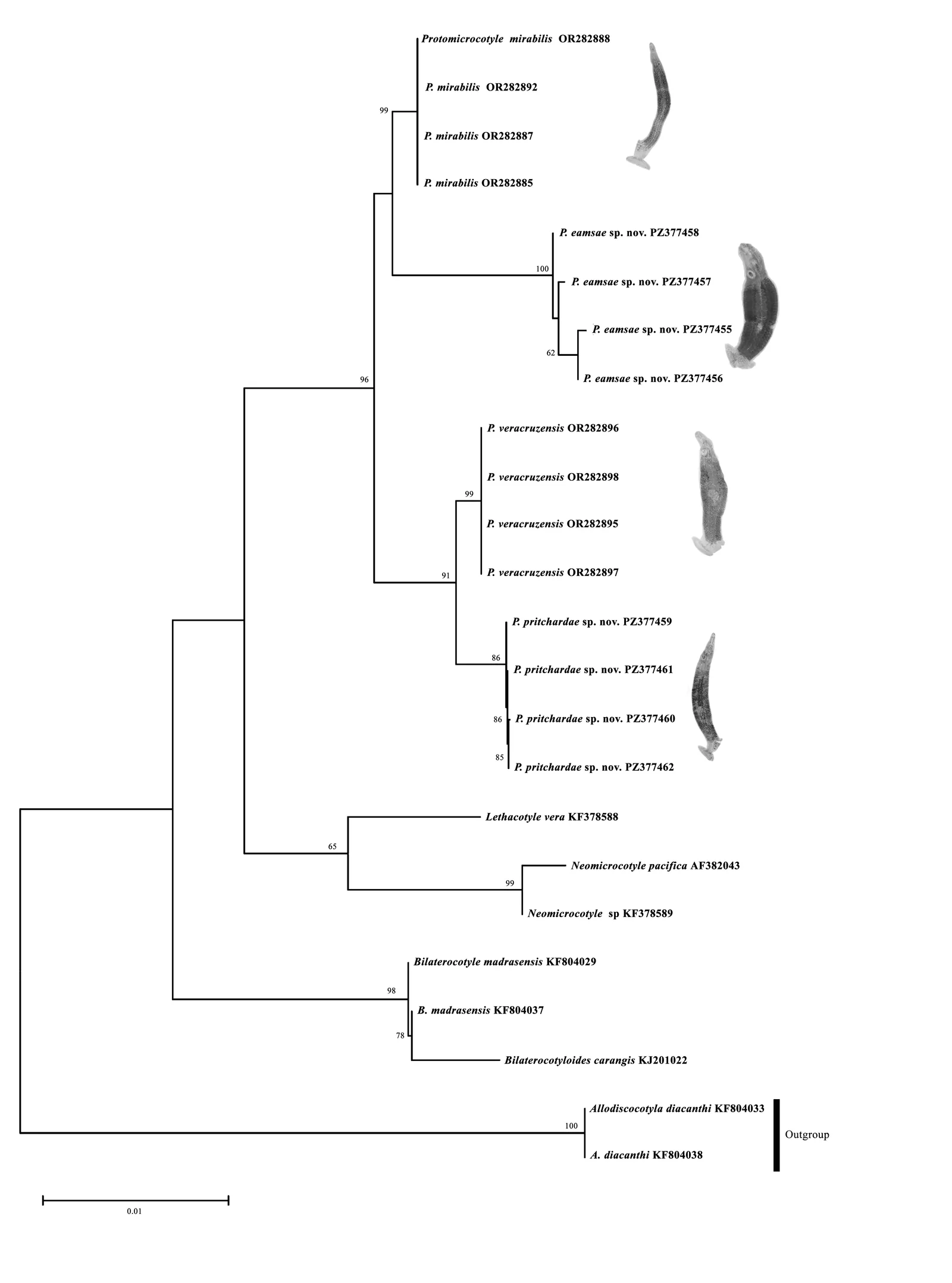
Neighbor Joining phenogram based on 28S rDNA gene sequences showing similarities between new species of *Protomicrocotyle* and other protomicrocotylids. Bootstrap values calculated for 1000 replications are indicated. Accession numbers from GenBank are given.

Species of *Protomicrocotyle* from the Gulf of Mexico form a cluster (support of 100%) in the cluster analysis (Figure 12). The specimens of *P*.*mirabilis* form a group with statistical support of 100%. The second cluster is comprised of *P*.*veracruzensis*, *P*.*eamsae* sp. nov. and *P*.*pritchardae* sp. nov., with statistical support of 98% (Figure 12). The topology displayed in the NJ dendrogram for 28S rDNA is similar as that of CO1 mtDNA, but *P*.*mirabilis* and *P*.*eamsae* sp. nov. are clustered together (Figure 13) and *P*.*veracruzensis* and *P*.*pritchardae* form a second group with statistical support of 91% (Figure 13).

## Discussion

The two new species of *Protomicrocotyle* described in this study have the morphological characteristics typical of the genus, including: an asymmetrical haptor with four Gastrocotylidae-type clamps in a longitudinal row on the side opposite to the vaginal vestibule; the haptoral lappet is transversely elongate (wider than long), armed with two pairs of anchors (larger anchors positioned laterally with smaller anchors between the larger ones), and one pair of hooks (located between the smaller anchors); the testes are relatively numerous, with variations in shape, size, and arrangement, but they always are anterior to the female complex; the MCO is bulbous, may be muscular, and is provided with a crown of numerous spines; vagina opening ventrally to the right or left, posterior to genital pore, with numerous spines of different shapes; vitellaria extends lateral and dorsal to ceca and eggs with filament at each pole (Bravo-Hollis, 1966; Bravo-Hollis, 1979; Kritsky et al., 2011; Lebedev, 1986; Ramalingam, 1960; Ramírez-Cruz et al., 2023; Wahl, 1972; Yamaguti, 1953).

Descriptions of monogeneans that include morphological data and genetic analyses has become a common practice (Kritsky & Martin, 2023; Rahmouni et al., 2023; Ramírez-Cruz et al., 2023). Both types of data individually have specific limitations, but together they provide more accurate taxonomic support. The results of the multivariate analyses allowed differentiation and separation of the specimens: *Protomicrocotyle eamsae* sp. nov., and *P. pritchardae* sp. nov., collected in *C*. *hippos* from the locality of Tecolutla, Veracruz from each other and from other specimens collected in the Gulf of Mexico. The main morphological characteristics that separate them are the length of the body, with *Protomicrocotyle eamsae* sp. nov., being smaller (1682) than *P. pritchardae* sp. nov. (4117) and the number of testes (39 vs. 60), the shape (elongated horizontally vs. subspherical), and the arrangement of the testes (two fields, with a row in each field vs. a single field). The aforementioned morphological characteristics, when considered in conjunction with the other measured variables (Tables 2, 3) and the results of the statistical analyses (Figures 11A, 11B, 11C; Tables 4, 5), support the separation of these specimens into distinct groups, each of then differentiated from the rest of the species under study (Figures 11A, 11B).

In this study, a novel partial sequence of CO1 mtDNA and 28S rDNA were generated for species within *Protomicrocotyle*. The partial sequences of each gene were employed in the analyses of genetic distance and for the analysis of phylogenetic inference using Neighbor Joining, based on genetic distances (Table 1). The newly-generated sequences provided new information about the intra- and interspecific variation of this group of monogeneans. The degree of intraspecific variation observed in the CO1 mtDNA between *P*.*mirabilis* was 1.03%–2.01% (Ramírez-Cruz et al., 2023), *P*.*veracruzensis* was 0.25%–0.75% (Ramírez-Cruz et al., 2023), *P. eamsae* sp. nov. was 0.0% –0.25%, and *P. pritchardae* sp. nov. was 0.0%–0.26% (Table 7A). These values are relatively low in comparison to those observed within other species of Microcotylidae: for example, 4.5% (Mladineo et al., 2009), 4%–7% (Farjallah et al., 2023), and in Mazocraeidae, 5.6% (Yan et al., 2016).

**Table 7:** Percentage of average genetic variation (uncorrected *p-distances*), based on cytochrome *b* partial sequences (**A**) and the conserved ribosomal regions of the *28S rDNA* data (**B**), observed within and between pairs of *Protomicrocotyle eamsae* sp. nov., and *Protomicrocotyle* *pritchardae* sp. nov., and other species of Protomicrocotylidae and Allodiscocotylidae included in the analyses.

**A**																	
Sequence number	KF804045		KF804046		KF804041		KF804043		OR282823		OR282824		OR282831		OR282832		OR282833
KF804045																	
KF804046	3.55%																
KF804041	19.86%		22.34%														
KF804043	18.09%		20.21%		12.41%												
OR282823	20.21%		23.05%		16.31%		14.89%										
OR282824	20.92%		23.76%		16.31%		14.89%		1.05%								
OR282831	19.86%		22.70%		16.67%		14.54%		0.76%		1.05%						
OR282832	20.92%		23.76%		16.31%		15.60%		1.53%		0.79%		1.27%				
OR282833	20.57%		23.40%		15.60%		16.31%		9.44%		9.45%		9.18%		9.95%		
OR282834	20.92%		23.76%		15.25%		16.67%		9.67%		9.45%		9.41%		10.18%		0.51%
OR282835	20.92%		23.76%		15.25%		16.67%		9.41%		9.19%		9.16%		9.92%		0.26%
OR282836	20.92%		23.76%		15.25%		16.67%		9.67%		9.45%		9.41%		10.18%		0.51%
PZ380651*	21.99%		24.82%		16.31%		17.38%		8.14%		7.87%		7.89%		8.65%		3.83%
PZ380652*	21.28%		24.11%		17.02%		16.67%		2.73%		2.81%		3.00%		3.26%		7.96%
PZ380653*	21.28%		24.11%		17.02%		16.31%		2.23%		2.81%		2.50%		3.26%		7.46%
PZ380654*	21.63%		24.47%		16.67%		16.67%		2.73%		2.81%		3.00%		3.26%		7.96%
PZ380648*	20.57%		23.40%		15.25%		15.96%		8.44%		7.93%		7.75%		8.77%		4.73%
PZ380649*	20.57%		23.40%		14.89%		16.31%		8.68%		8.18%		8.00%		9.02%		4.98%
PZ380650*	20.57%		23.40%		15.25%		15.96%		8.44%		7.93%		7.75%		8.77%		4.73%
	OR282834		OR282835		OR282836		**PZ380651** *		PZ380652*		PZ380653*		PZ380654*		PZ380648*		PZ380649*
OR282835	0.25%																
OR282836	0.51%		0.25%														
PZ380651*	3.82%		3.56%		3.82%												
PZ380652*	3.56%		3.31%		3.56%		***0.25%***										
PZ380653*	3.56%		3.31%		3.56%		***0.25%***		***0.00%***								
PZ380654*	3.82%		3.56%		3.82%		***0.00%***		***0.25%***		***0.25%***						
PZ380648*	1.28%		1.02%		1.28%		4.60%		4.35%		4.35%		4.60%				
PZ380649*	1.27%		1.02%		1.27%		4.58%		4.33%		4.33%		4.58%		***0.00%***		
PZ380650*	1.53%		1.28%		1.53%		4.85%		4.59%		4.59%		4.85%		***0.26%***		***0.26%***
**B**																	
Sequence number		KF804033		KF804038		KF804029		KF804037		KJ201022		AF382043		KF378589		KF378588	
KF804033																	
KF804038		0.00%															
KF804029		5.25%		5.25%													
KF804037		5.25%		5.25%		0.00%											
KJ201022		5.29%		5.29%		0.44%		0.44%									
AF382043		6.06%		6.06%		2.42%		2.42%		2.94%							
KF378589		5.93%		5.93%		2.20%		2.20%		2.79%		0.14%					
KF378588		6.21%		6.21%		3.03%		3.03%		3.38%		1.79%		1.65%			
OR282885		5.25%		5.25%		2.55%		2.55%		3.23%		2.69%		2.48%		2.20%	
OR282887		5.26%		5.26%		2.56%		2.56%		3.24%		2.69%		2.48%		2.21%	
OR282888		5.26%		5.26%		2.56%		2.56%		3.24%		2.70%		2.48%		2.21%	
OR282892		5.27%		5.27%		2.56%		2.56%		3.23%		2.70%		2.48%		2.20%	
OR282895		5.38%		5.38%		2.96%		2.96%		3.67%		2.82%		2.62%		2.48%	
OR282896		5.38%		5.38%		2.96%		2.96%		3.67%		2.82%		2.62%		2.48%	
OR282897		5.38%		5.38%		2.96%		2.96%		3.67%		2.82%		2.62%		2.48%	
OR282898		5.41%		5.41%		2.97%		2.97%		3.68%		2.83%		2.62%		2.48%	
PZ377455*		6.22%		6.22%		3.51%		3.51%		3.52%		3.78%		2.89%		2.62%	
PZ377456*		6.36%		6.36%		3.65%		3.65%		3.67%		3.65%		2.75%		2.48%	
PZ377457*		6.11%		6.11%		3.39%		3.39%		3.52%		3.66%		2.89%		2.62%	
PZ377458*		6.24%		6.24%		3.52%		3.52%		3.67%		3.52%		2.75%		2.48%	
PZ377459*		5.59%		5.59%		3.13%		3.13%		3.82%		3.27%		3.03%		2.89%	
PZ377460*		5.52%		5.52%		3.09%		3.09%		3.82%		3.23%		3.03%		2.89%	
PZ377461*		5.59%		5.59%		3.13%		3.13%		3.82%		3.27%		3.03%		2.89%	
PZ377462*		5.59%		5.59%		3.13%		3.13%		3.82%		3.27%		3.03%		2.89%	
		OR282885		OR282887		OR282888		OR282892		OR282895		OR282896		OR282897		OR282898	
OR282887		0.00%															
OR282888		0.00%		0.00%													
OR282892		0.00%		0.00%		0.00%											
OR282895		0.81%		0.81%		0.81%		0.81%									
OR282896		0.81%		0.81%		0.81%		0.81%		0.00%							
OR282897		0.81%		0.81%		0.81%		0.81%		0.00%		0.00%					
OR282898		0.81%		0.81%		0.81%		0.81%		0.00%		0.00%		0.00%			
PZ377455*		1.22%		1.22%		1.22%		1.22%		1.89%		1.89%		1.89%		1.89%	
PZ377456*		1.08%		1.08%		1.08%		1.08%		1.76%		1.76%		1.76%		1.76%	
PZ377457*		1.08%		1.09%		1.09%		1.08%		1.76%		1.76%		1.76%		1.76%	
PZ377458*		0.95%		0.95%		0.95%		0.95%		1.63%		1.63%		1.63%		1.63%	
PZ377459*		0.95%		0.95%		0.95%		0.95%		0.41%		0.41%		0.41%		0.41%	
PZ377460*		0.94%		0.94%		0.94%		0.94%		0.40%		0.40%		0.40%		0.40%	
PZ377461*		0.95%		0.95%		0.95%		0.95%		0.41%		0.41%		0.41%		0.41%	
PZ377462*		0.95%		0.95%		0.95%		0.95%		0.41%		0.41%		0.41%		0.41%	
		PZ377455*		PZ377456*		PZ377457*		PZ377458*		PZ377459*		PZ377460*		PZ377461*		PZ377462*	
PZ377456*		***0.13%***															
PZ377457*		***0.00%***		***0.13%***													
PZ377458*		***0.13%***		***0.00%***		***0.13%***											
PZ377459*		1.49%		1.35%		1.49%		1.35%									
PZ377460*		2.14%		2.01%		2.02%		1.88%		***0.00%***							
PZ377461*		1.62%		1.48%		1.62%		1.48%		***0.00%***		***0.00%***					
PZ377462*		1.62%		1.48%		1.62%		1.48%		***0.00%***		***0.00%***		***0.00%***			

* New sequences of *Protomicrocotyle eamsae* sp. nov., and *Protomicrocotyle* *pritchardae* sp. nov.

The **bold** and *italic* values correspond to the intraspecific data of the new species of *Protomicrocotyle*.

The degree of interspecific variation between *P. mirabilis* and *P. veracruzensis* is high (9.21%–10.53%), but the difference between *P. veracruzensis* and *P*.*pritchardae* sp. nov. are medium low (1.02%–1.53%). In contrast, the degree of variation between *P*.*mirabilis* and *P*.*eamsae* sp. nov. is relatively high (7.61%–8.65) (Table 7A). The degree of interspecific variation between *P*.*eamsae* sp. nov. and *P*.*pritchardae* sp. nov. is 4.33%–4.85% (Table 7A). However, lower levels of interspecific variation have been reported between species of *Microcotyle*, with figures of 0.7% (Lablack et al., 2022), 4.5% (Ayadi et al., 2017; Víllora-Montero et al., 2020), and 6.1% (Ono et al., 2020).

The phenogram of NJ revealed that the genetic sequences of *P*.*mirabilis*, *P*.*veracruzensis*, *P*.*eamsae* sp. nov. and *P*.*pritchardae* sp. nov. form a cluster with high statistical support (Figure 12). Kamio & Nitta (2022) included the analysis of partial CO1 mtDNA sequences of the species *Bilaterocotyle madrasensis* Radha, 1966 and *Bilaterocotyloides carangis* Ramalingam, 1961 of the family Protomicrocotylidae, although the relationships remain unresolved in their Bayesian inference tree. When the data is available, it would be advisable to incorporate additional sequences and undertake a more inclusive phylogenetic analysis of these families.

Intraspecific variation in 28S rDNA of *P*. *mirabilis* was 0% to 0.14% (Ramírez-Cruz et al., 2023); *P*.*veracruzensis* was 0% (Ramírez-Cruz et al., 2023), *P*.*eamsae* sp. nov. were 0% to 0.13%, and *Protomicrocotyle pritchardae* sp. nov. was 0% (Table 7B). The sequences of 28S rDNA of species of *Protomicrocotyle* were grouped together with high support values (96%) (Figure 13). The combination of genetic sequences, morphological comparisons and morphometric analyses provides supplementary information that facilitates the differentiation of taxa. In the present study, the data obtained from both markers is supplementary to the morphological data, thereby corroborating the distinction of the two novel species of *Protomicrocotyle*.

Comparative sequence analyses of the species of monogeneans showed that both partial CO1 mtDNA and 28S rDNA genes can be successfully used for species identification (Kamio et al., 2023; Pinacho-Pinacho et al., 2021; Rahmouni et al., 2017; Ramírez-Cruz et al., 2023; Torres-Carrera et al., 2020; Zago et al., 2021) and for phylogenetic inference (Justine et al., 2013; Kamio & Nitta, 2022; Tambireddy et al., 2016). The available molecular sequence information for species of Mazocraeidea is insufficient, necessitating the accumulation of molecular studies for phylogenetic analysis with morphological and molecular data (Tambireddy et al., 2016). In Protomicrocotylidae, data is available only for *Bilaterocotyle madrasensis*, *Bilaterocotyloides carangis*, *Neomicrocotyle pacifica*, *Lethacotyle vera*, *Protomicrocotyle mirabilis*, *P*. *veracruzensis*, *P*.*eamsae* sp. nov., and *P*.*pritchardae* sp. nov., and *Neomicrocotyle* sp. This information about species of *Protomicrocotyle* was produced as a result of Ramírez-Cruz et al. (2023) and this study (Table 1).

Members of the family of Carangidae are usual hosts for monogenean parasites (Martínez-Flores et al., 2023; Montoya-Mendoza et al., 2017, 2021; Ramírez-Cruz et al., 2023; Vianna et al., 2020; Violante-González et al., 2019). Several species of Protomicrocotylidae commonly infect the gills of carangid fish (Bravo-Hollis, 1966, 1979, 1989; Caballero y Caballero & Bravo-Hollis, 1965; Kritsky et al., 2011; Mendoza-Garfias et al., 2017; Ramírez-Cruz et al., 2023; Yamaguti, 1963). In the Gulf of Mexico, the most common species of *Caranx* are: *C. hippos*, *C. crysos* (Mitchill, 1815), and *C. latus* (López-Herrera et al., 2021; Nelson et al., 2016). *Caranx hippos* was considered to have been sufficiently surveyed throughout much of its distribution range (Kritsky et al., 2011), but the findings of Ramírez-Cruz et al. (2023) and this study, have shown that there is much more to learn from the parasitofauna of this species of host.

This work adds two new species to the list of protomicrocotylids from Mexico and the increases the record of species of monogeneans from *C*.*hippos* to 19 species (including the two new species): *Axine* sp., *Ahpua piscicola* Caballero y Caballero & Bravo-Hollis, 1973; *Allopyragraphorus caballeroi* (Zerecero, 1960) Yamaguti, 1963; *All.* *hippos* (Hargis, 1956) Yamaguti, 1963; *All. winteri* (Caballero y Caballero & Bravo-Hollis, 1965) Bravo-Hollis & Salgado-Maldonado, 1983; *Cemocotyle carangis* (MacCallum, 1913) Sproston, 1946 (Mendoza-Garfias et al., 2017); *Ce. noveboracensis* Price, 1962 (Luque & Alves, 2001; Mendoza-Garfias et al., 2017); *Cemocotylella elongata* (Meserve, 1938) Price, 1962 (Mendoza-Garfias et al., 2017); *Lethacotyle* sp. (Odum & Amuzie, 2021); *Neomicrocotyle pacifica*; *Pseudomazocraes monsivaisae* Caballero y Caballero & Bravo Hollis, 1955; *Ps. riojai* (Caballero y Caballero & Bravo-Hollis, 1963) Lebedev, 1970 (Caballero y Caballero & Bravo-Hollis, 1963); *Ps. selene* Hargis, 1957, *P. mirabilis* (Kritsky et al., 2011; Ramírez-Cruz et al., 2023); (Kritsky et al., 2011; Ramírez-Cruz et al., 2023) *P. veracruzensis* (Ramírez-Cruz et al., 2023); *Salinacotyle mexicana* (Caballero y Caballero & Bravo-Hollis, 1963) Lebedev, 1984; and *Zeuxapta seriolae* (Meserve, 1938) Price, 1962 (Mendoza-Garfias et al., 2017). To this list, *P. eamsae* sp. nov. and, *P*.*pritchardae* sp. nov. can now be added.

In the past, *C. hippos* was considered to be distributive in both the Pacific and Atlantic Oceans (Torres-Orozco & Pérez-Hernández, 2009). The distribution of *C. hippos* is now recognized as being restricted to the Atlantic Ocean. *Caranx fischeri* Smith-Vaniz & Carpenter, 2007 has a distribution in the Eastern Atlantic, the Mediterranean, and Ascension Island. *Caranx caninus* and *C. caballus* Günther, 1868 are distributed along the Eastern Pacific costs of North and South America (Mair et al., 2012; Smith-Vaniz & Carpenter, 2007). The original host of *Ah. piscicola*, *All. caballeroi*, *N. pacifica*, *Ps. monsivaisae*, *Ps. riojai* and *S. mexicana* were reported as *C. hippos* in Pacific waters; these species are now considered to be parasites of either *C. caballus* or *C. caninus* (Pulido-Flores et al., 2015), but the original reports have not been brought up-to-date.

## Data Availability

The data supporting the findings of this study will be available upon request.
